# Supplementary material for: TRIP12 structures reveal HECT E3 formation of K29 linkages and branched ubiquitin chains
Source: Nat Struct Mol Biol. 2025 May 26;32(9):1766–75. doi: 10.1038/s41594-025-01561-1 (PMC12440805; doi:10.1038/s41594-025-01561-1)
Supplement: Supplementary file 1 — Supplementary Note 1, Figs. 1–5 and Table 1. [file 41594_2025_1561_MOESM1_ESM.pdf]

nature structural & molecular biology

Supplementary information https://doi.org/10.1038/s41594-025-01561-1

TRIP12 structures reveal HECT E3 formation of K29 linkages and branched ubiquitin chains

In the format provided by the authors and unedited

Supplementary Information

Supplementary Note 1: Ub mutant synthesis

Solid Phase Peptide Synthesis (SPPS)

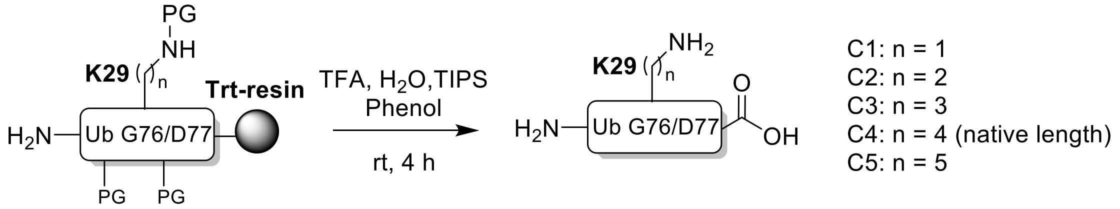

The Ub (mutant) peptide sequences were obtained on a Syro II MultiSyntech Automated Peptide synthesizer using standard 9-fluorenylmethoxycarbonyl (Fmoc) based solid phase peptide chemistry on trityl-resin as described in literature on a 10 or 20 mmol scale.[1] The ubiquitin on trityl resin was dissolved in a mixture of TFA/H_2O/TIPS/phenol (90.5/5/2/2.5 v/v/v/v; 4 mL) and stirred with an orbital shaker for 4 hours at rt towards global deprotection. The protein was precipitated with ice-cold Et_2O/*n*-pentane (3/1; v/v; 40 mL). The precipitated protein was isolated by centrifugation (4000 rpm, 4°C, 6 min) and washed by three cycles of resuspension in ice-cold diethyl ether and centrifugation. The pellet was dissolved in H_2O/MeCN/FA (75/24/1; v/v/v; 20 mL) and lyophilized. The crude product was purified using RP-HPLC and appropiate fractions as judged by LC-MS were pooled and lyophilized to afford the ubiquitin peptide as a white solid.
<u>Ubiquitin K29 D77 C1:</u> ES MS+ (amu) calcd: 8621, found 8621. LCMS: R_t = 3.08 min.
<u>Ubiquitin K29 D77 C2:</u> ES MS+ (amu) calcd: 8635, found 8635. LCMS: R_t = 3.06 min.
<u>Ubiquitin K29 D77 C3:</u> ES MS+ (amu) calcd: 8649, found 8649. LCMS: R_t = 3.07 min.
<u>Ubiquitin K29 D77 C4:</u> ES MS+ (amu) calcd: 8663, found 8663. LCMS: R_t = 3.07 min.
<u>Ubiquitin K29 D77 C5:</u> ES MS+ (amu) calcd: 8677, found 8677. LCMS: R_t = 3.09 min.

LC-MS measurements were performed on a system equipped with a Waters 2795 Seperation Module (Alliance HT), Waters 2996 Photodiode Array Detector (190-750nm), Phenomenex Kinetex C18 (2.1x100, 2.6 μm) column and LCTTM Orthogonal Acceleration Time of Flight Mass Spectrometer. Samples were run using 3 mobile phases: A (H_2O), B (acetonitrile) and constant 4% of C (2.5% formic acid in 1:1 H_2O:acetonitrile) at a flow rate of 500 μL/min; gradient: 0–0.5 min, 2 % B; 0.5–7.5 min, → 96 % B; 7.5–8.0 min 96% B; 8.0–8.1 min, → 2 % B; 8.1–10 min 2% B. Data processing was performed using Waters MassLynx Mass Spectrometry Software 4.1 (deconvolution with MaxEnt1 function).
RP-HPLC purification was performed on a Waters XBridge BEH C18 Prep Column 30x150 5μm. Column Mobile phases: A=1% aq. TFA and B=CH_3CN. Flow rate=37.5 mL/min. Gradient: 5→45%B over 13 min. For the K29 C5 mutants an additional RP-HPLC purification was performed on a Waters XBridge C18 4.6x150 5 μm. Column Mobile phases: A=0.05% aq. TFA and B=0.05% TFA in CH_3CN. Flow rate = 6.5 mL/min. Gradient: 25→35%B over 10 min.

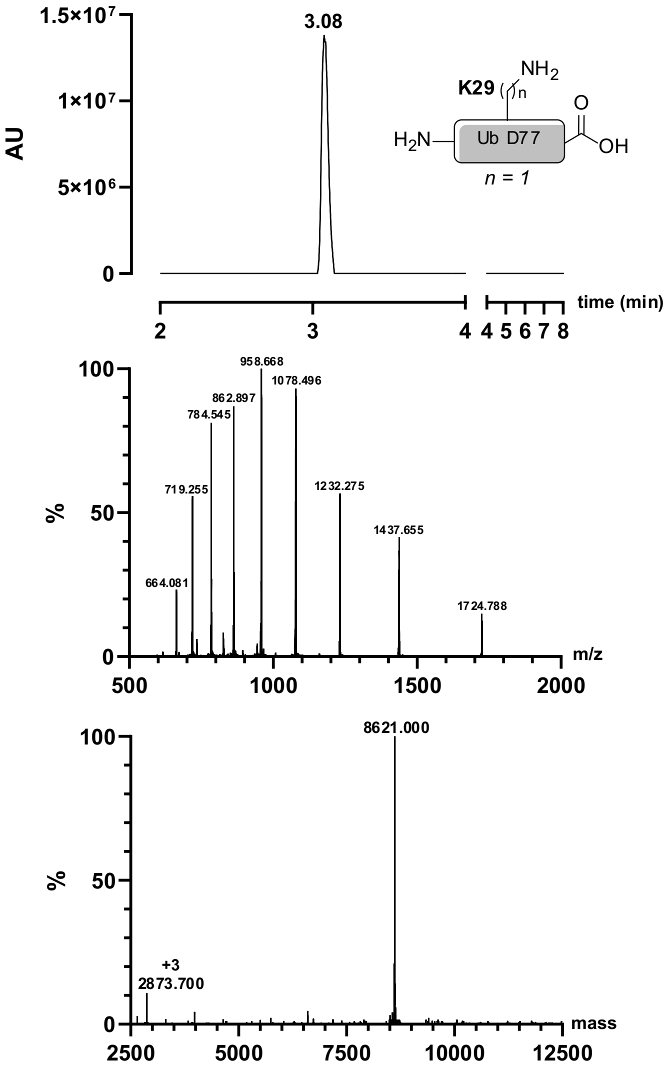

Figure S1. *Ubiquitin D77 K29 C1. Diode array chromatogram (top). MS spectrum (middle). Deconvoluted mass of product peak (bottom). ESI [M+H]$^+$ Expected: 8621, found 8621.*

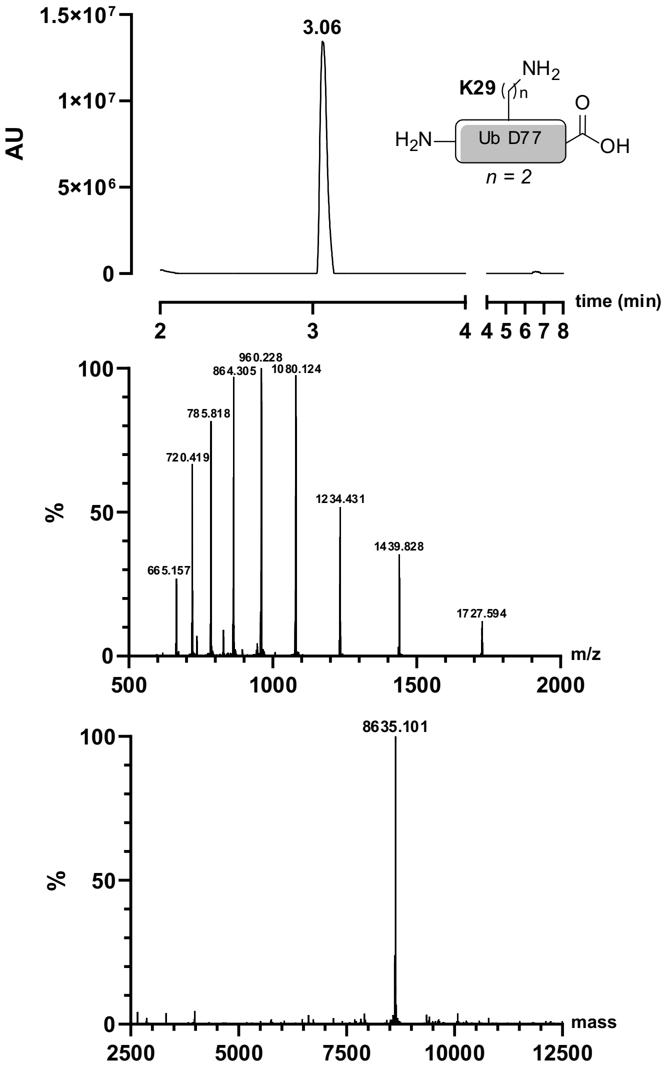

Figure S2. *Ubiquitin D77 K29 C2. Diode array chromatogram (top). MS spectrum (middle). Deconvoluted mass of product peak (bottom). ESI $[M+H]^+$ Expected: 8635, found 8635.*

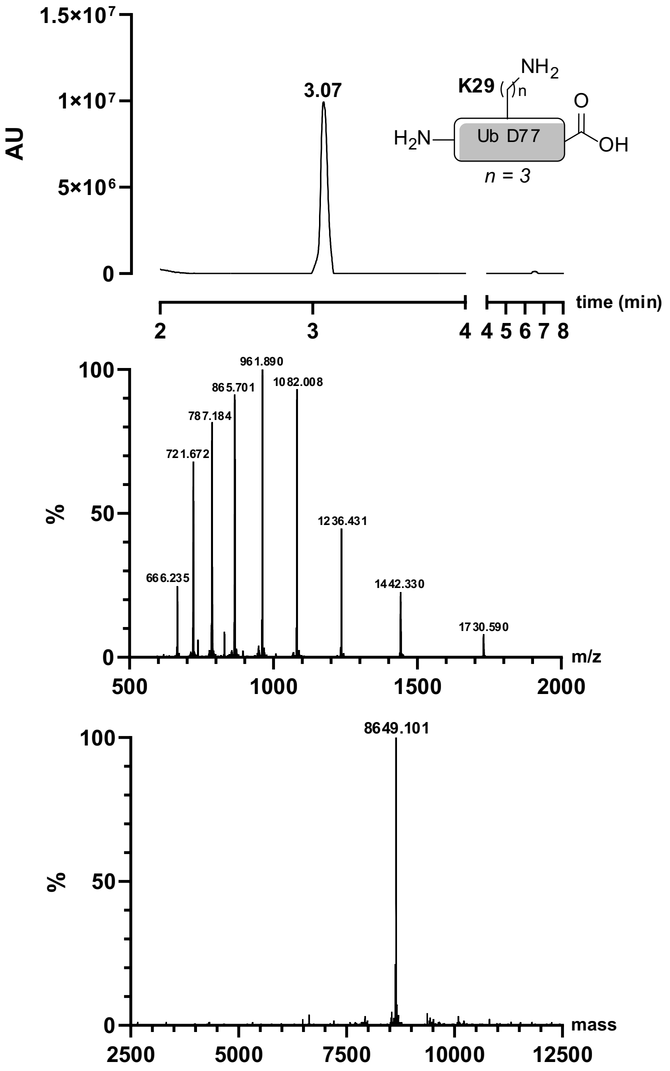

Figure S3. *Ubiquitin D77 K29 C3. Diode array chromatogram (top). MS spectrum (middle). Deconvoluted mass of product peak (bottom). ESI [M+H]$^+$ Expected: 8649, found 8649.*

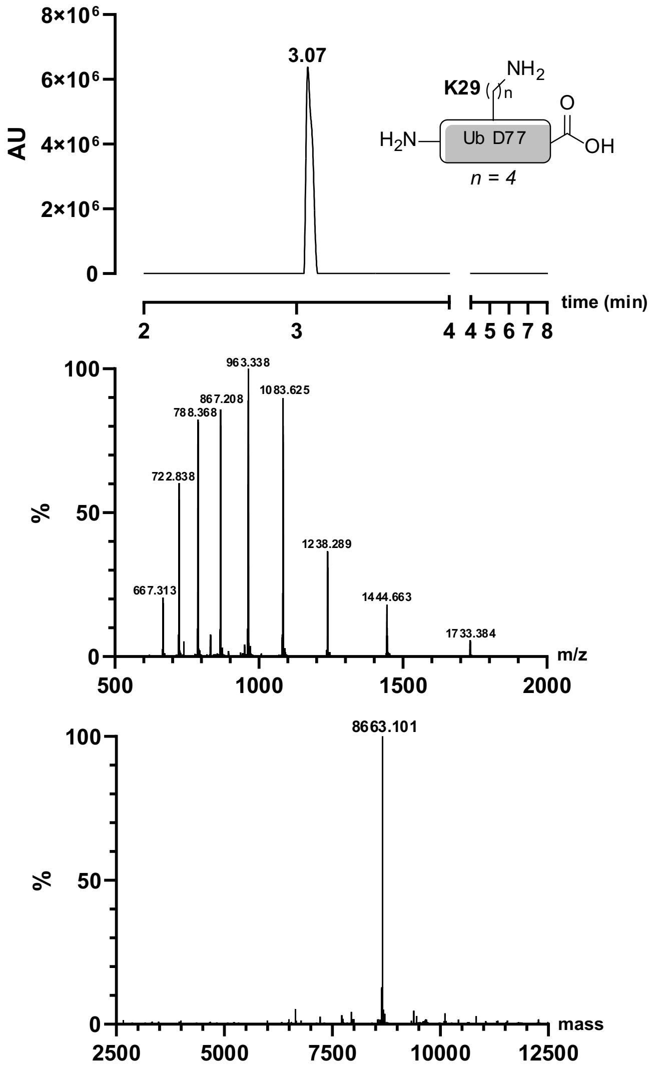

Figure S4. *Ubiquitin D77 K29 C4. Diode array chromatogram (top). MS spectrum (middle). Deconvoluted mass of product peak (bottom). ESI* $[M+H]^+$ *Expected: 8663, found 8663.*

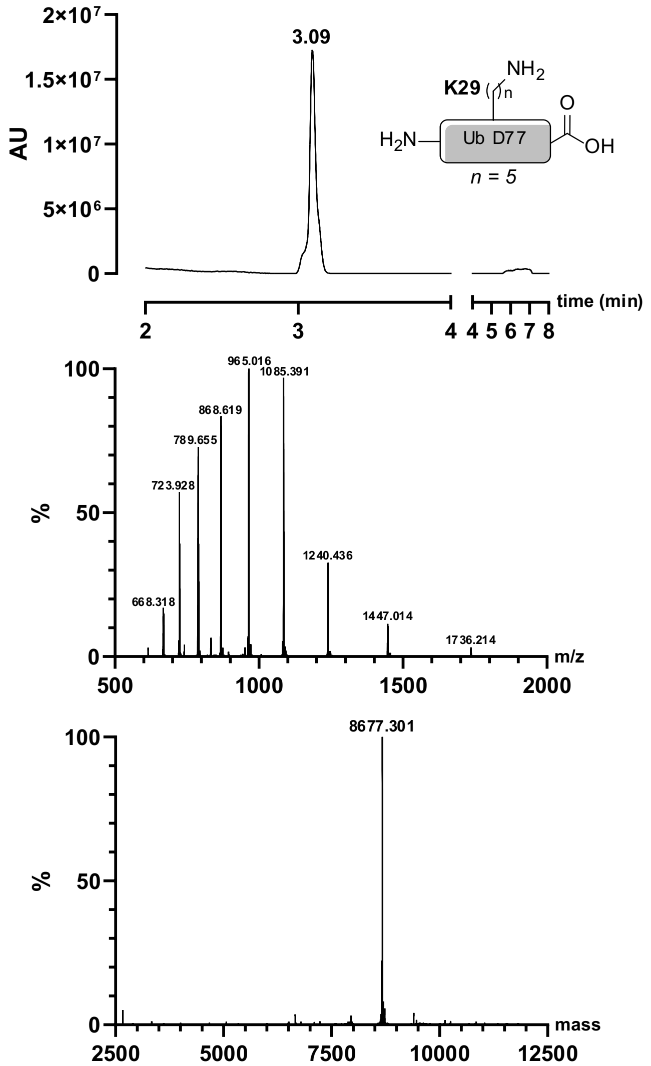

Figure S5. *Ubiquitin D77 K29 C5. Diode array chromatogram (top). MS spectrum (middle). Deconvoluted mass of product peak (bottom). ESI $[M+H]^+$ Expected: 8677, found 8677.*

Supplementary Table 1: Oligonucleotides used for Cloning

Description	Sequence	Purpose
pLIB_f	tgaaaggcctacgtcgacg	Vector linearization for Gibson Assembly
oLIB_TEV_r	ggatccctgaaaatacaggttttc	Vector linearization for Gibson Assembly
TRIP12_forpLIB_f	gtattttcagggatccatgtccaaccggcctaataacaatcc	Insert generation for Gibson Assembly
TRIP12dN_forpLIB_f	gtattttcagggatccacaattggaagtggagctagttctaagg	Insert generation for Gibson Assembly
TRIP12_forpLIB_r	cgtcgacgtaggcctttcaggaaagatggaacgactgctgc	Insert generation for Gibson Assembly
TRIP12_V514W_f	GAGATGTGTCAGTTACTGTGGATGGGAAATGAGGAGAC	Quikchange mutagenesis
TRIP12_V514W_r	GTCTCCTCATTTCCCATCCACAGTAACTGACACATCTC	Quikchange mutagenesis
TRIP12_I584A_f	CAAGTTATTCAGTGTGCTGATGTGGCAGAGCAG	Quikchange mutagenesis
TRIP12_I584A_r	CTGCTCTGCCACATCAGCACACTGAATAACTTG	Quikchange mutagenesis
TRIP12_F620A_f	GCTTGCTGTACCTAGAAGCTTTCAGCATAAATGCCC	Quikchange mutagenesis
TRIP12_F620A_r	GGGCATTTATGCTGAAAGCTTCTAGGTACAGCAAGC	Quikchange mutagenesis
TRIP12_Y1668A_E1671A_f	GCCATGTTAGAAATCCAGGCTGAAAATGCTGTTGGTACAGGTCTTGG	Quikchange mutagenesis
TRIP12_Y1668A_E1671A_r	CCAAGACCTGTACCAACAGCATTTTCAGCCTGGATTTCTAACATGGC	Quikchange mutagenesis
TRIP12_H1935A_f	GAATGCTGTAGGCCTGATGCTGGTTATACTCATGAC	Quikchange mutagenesis
TRIP12_H1935A_r	GTCATGAGTATAACCAGCATCAGGCCTACAGCATTC	Quikchange mutagenesis
TRIP12_FL2000/1AA_f	CAGAAAACCCAGATGACGCTGCTCCCTCTGTAATGACTTG	Quikchange mutagenesis
TRIP12_FL2000/1AA_r	CAAGTCATTACAGAGGGAGCAGCGTCATCTGGGTTTTCTG	Quikchange mutagenesis
TRIP12_M2005A_f	GACTTCTTGCCCTCTGTAGCTACTTGTGTGAACTATC	Quikchange mutagenesis
TRIP12_M2005A_r	GATAGTTCACACAAGTAGCTACAGAGGGCAAGAAGTC	Quikchange mutagenesis
TRIP12_Y2010A_f	GTAATGACTTGTGTGAACGCTCTTAAGTTGCCGG	Quikchange mutagenesis
TRIP12_Y2010A_r	CCGGCAACTTAAGAGCGTTCACACAAGTCATTAC	Quikchange mutagenesis
TRIP12_K2012D_f	GACTTGTGTGAACTATCTTGACTTGCCGGACTATTCAAG	Quikchange mutagenesis
TRIP12_K2012D_r	CTTGAATAGTCCGGCAAGTCAAGATAGTTCACACAAGTC	Quikchange mutagenesis
TRIP12_I2028A_f	CGTGAAAAACTGTTGGCTGCAGCAAGAGAAGGG	Quikchange mutagenesis
TRIP12_I2028A_r	CCCTTCTCTTGCTGCAGCCAACAGTTTTTCACG	Quikchange mutagenesis
TRIP12_L2039D_f	CAGCAGTCGTTCCATGACTCCTGAAAGGCCTAC	Quikchange mutagenesis
TRIP12_L2039D_r	GTAGGCCTTTCAGGAGTCATGGAACGACTGCTG	Quikchange mutagenesis
TRIP12_L2039W_f	CAGCAGTCGTTCCATTGGTCCTGAAAGGCCTAC	Quikchange mutagenesis
TRIP12_L2039W_r	GTAGGCCTTTCAGGACCAATGGAACGACTGCTG	Quikchange mutagenesis
TRIP12_S2040A_f	CGTTCCATCTTGCTTGAAAGGCCTACGTC	Quikchange mutagenesis
TRIP12_S2040A_r	GACGTAGGCCTTTCAAGCAAGATGGAACG	Quikchange mutagenesis
TRIP12_S2040D_f	GCAGTCGTTCCATCTTGATTGAAAGGCCTACGTC	Quikchange mutagenesis
TRIP12_S2040D_r	GACGTAGGCCTTTCAATCAAGATGGAACGACTGC	Quikchange mutagenesis
TRIP12_S2040V_f	CAGTCGTTCCATCTTGTCTGAAAGGCCTACG	Quikchange mutagenesis
TRIP12_S2040V_r	CGTAGGCCTTTCAGACAAGATGGAACGACTG	Quikchange mutagenesis
TRIP12_S2040N_f	GTCGTTCCATCTTAACTGAAAGGCCTACGTC	Quikchange mutagenesis
TRIP12_S2040N_r	GACGTAGGCCTTTCAGTTAAGATGGAACGAC	Quikchange mutagenesis
TRIP12_S2040E_f	GTCGTTCCATCTTGAATGAAAGGCCTACGTCG	Quikchange mutagenesis

TRIP12_S2040E_r	CGACGTAGGCCTTTCATTCAAGATGGAACGAC	Quikchange mutagenesis
TRIP12_S2040L_f	CAGTCGTTCCATCTTCTGTGAAAGGCCTACGTCG	Quikchange mutagenesis
TRIP12_S2040L_r	CGACGTAGGCCTTTCACAGAAGATGGAACGACTG	Quikchange mutagenesis
TRIP12_S2040I_f	GTCGTTCCATCTTATCTGAAAGGCCTACGTCG	Quikchange mutagenesis
TRIP12_S2040I_r	CGACGTAGGCCTTTCAGATAAGATGGAACGAC	Quikchange mutagenesis
TRIP12_S2040Y_f	CGTTCCATCTTTACTGAAAGGCCTAC	Quikchange mutagenesis
TRIP12_S2040Y_r	GTAGGCCTTTCAGTAAAGATGGAACG	Quikchange mutagenesis
TRIP12_S2040H_f	CAGTCGTTCCATCTTCACTGAAAGGCCTACG	Quikchange mutagenesis
TRIP12_S2040H_r	CGTAGGCCTTTCAGTGAAGATGGAACGACTG	Quikchange mutagenesis
TRIP12_2041V_f	CGTTCCATCTTTCCGTCTGAAAGGCCTACGTC	Quikchange mutagenesis
TRIP12_2041V_r	GACGTAGGCCTTTCAGACGGAAAGATGGAACG	Quikchange mutagenesis
Ub_K29R_f	CGAAAACGTTAAGGCTCGTATTCAAGACAAGGAAGG	Quikchange mutagenesis
Ub_K29R_r	CCTTCCTTGTCTTGAATACGAGCCTTAACGTTTTCG	Quikchange mutagenesis
Ub_K48R_f	GATCTTTGCCGGTCGTCAGCTCGAGGACGGTAGAACG	Quikchange mutagenesis
Ub_K48R_r	CGTTCTACCGTCCTCGAGCTGACGACCGGCAAAGATC	Quikchange mutagenesis
UbHis_E16K_f	GACCATCACCCTCAAAGTTGAACCCTCGGATAC	Quikchange mutagenesis
UbHis_E16K_r	GTATCCGAGGGTTCAACTTTGAGGGTGATGGTC	Quikchange mutagenesis
UbHis_D21A_f	GAGGTTGAACCCTCGGCGACGATAGAAAATGTAAAG	Quikchange mutagenesis
UbHis_D21A_r	CTTTACATTTTCTATCGTCGCCGAGGGTTCAACCTC	Quikchange mutagenesis
UbHis_N25D_f	CGGATACGATAGAAGATGTAAAGGCCAAG	Quikchange mutagenesis
UbHis_N25D_r	CTTGGCCTTTACATCTTCTATCGTATCCG	Quikchange mutagenesis
UbHis_R42A_f	CTCCTGATCAGCAGGCGCTGATCTTTGCTGGC	Quikchange mutagenesis
UbHis_R42A_r	GCCAGCAAAGATCAGCGCCTGCTGATCAGGAG	Quikchange mutagenesis
UbHis_R42E_f	CTCCTGATCAGCAGGAACTGATCTTTGCTGG	Quikchange mutagenesis
UbHis_R42E_r	CCAGCAAAGATCAGTTCCTGCTGATCAGGAG	Quikchange mutagenesis
UbD77_E16K_f	GTAAAACCATAACTCTAAAAGTTGAACCATCCGATAC	Quikchange mutagenesis
UbD77_E16K_r	GTATCGGATGGTTCAACTTTTAGAGTTATGGTTTTAC	Quikchange mutagenesis
UbD77_D21A_f	GAAGTTGAACCATCCGCGACCATCGAAAACG	Quikchange mutagenesis
UbD77_D21A_r	CGTTTTCGATGGTCGCGGATGGTTCAACTTC	Quikchange mutagenesis
UbD77_N25D_f	CATCCGATACCATCGAAGATGTTAAGGCTAAAATTCAAG	Quikchange mutagenesis
UbD77_N25D_r	CTTGAATTTTAGCCTTAACATCTTCGATGGTATCGGATG	Quikchange mutagenesis
UbD77_R42A_f	CCACCTGATCAACAAGCGTTGATCTTTGCCGG	Quikchange mutagenesis
UbD77_R42A_r	CCGGCAAAGATCAACGCTTGTTGATCAGGTGG	Quikchange mutagenesis
UbD77_R42E_f	CCACCTGATCAACAAGAATTGATCTTTGCCG	Quikchange mutagenesis
UbD77_R42E_r	CGGCAAAGATCAATTCTTGTTGATCAGGTGG	Quikchange mutagenesis

References

(1) Farid El Oualid; Remco Merkx; Reggy Ekkebus; Hameed, D. S.; Smit, J. J.; Annemieke de Jong; Henk Hilkmann; Sixma, T. K.; Huib Ovaa. Chemical Synthesis of Ubiquitin, Ubiquitin-Based Probes, and Diubiquitin. *Angewandte Chemie* **2010**, *49* (52), 10149–10153. https://doi.org/10.1002/anie.201005995.
