# Supplementary material for: TRIP12 structures reveal HECT E3 formation of K29 linkages and branched ubiquitin chains
Source: Nat Struct Mol Biol. 2025 May 26;32(9):1766–75. doi: 10.1038/s41594-025-01561-1 (PMC12440805; doi:10.1038/s41594-025-01561-1)
Supplement: Supplementary file 2 — Reporting Summary [file 41594_2025_1561_MOESM2_ESM.pdf]

nature portfolio

Corresponding author(s): Brenda A. Schulman

Last updated by author(s): Mar 18, 2025

Reporting Summary

Nature Portfolio wishes to improve the reproducibility of the work that we publish. This form provides structure for consistency and transparency in reporting. For further information on Nature Portfolio policies, see our Editorial Policies and the Editorial Policy Checklist.

Statistics

For all statistical analyses, confirm that the following items are present in the figure legend, table legend, main text, or Methods section.

n/a	Confirmed	
☐	☒	The exact sample size (n) for each experimental group/condition, given as a discrete number and unit of measurement
☐	☒	A statement on whether measurements were taken from distinct samples or whether the same sample was measured repeatedly
☒	☐	The statistical test(s) used AND whether they are one- or two-sided<br>*Only common tests should be described solely by name; describe more complex techniques in the Methods section.*
☒	☐	A description of all covariates tested
☒	☐	A description of any assumptions or corrections, such as tests of normality and adjustment for multiple comparisons
☒	☐	A full description of the statistical parameters including central tendency (e.g. means) or other basic estimates (e.g. regression coefficient) AND variation (e.g. standard deviation) or associated estimates of uncertainty (e.g. confidence intervals)
☒	☐	For null hypothesis testing, the test statistic (e.g. F, t, r) with confidence intervals, effect sizes, degrees of freedom and P value noted<br>*Give P values as exact values whenever suitable.*
☒	☐	For Bayesian analysis, information on the choice of priors and Markov chain Monte Carlo settings
☒	☐	For hierarchical and complex designs, identification of the appropriate level for tests and full reporting of outcomes
☒	☐	Estimates of effect sizes (e.g. Cohen's d, Pearson's r), indicating how they were calculated

Our web collection on statistics for biologists contains articles on many of the points above.

Software and code

Policy information about availability of computer code

Data collection	Cryo-EM data acquisition: SerialEM v4.1; Gel & blot imaging: Amersham Imager 600; Fluorescent gel scanning: Amersham Typhoon
Data analysis	Cryo-EM data processing: CryoSparc v4.4.0, RELION v5.0 beta, Gautomatch v0.56, CTFFIND v4.1; Structure Analysis and Visualization: ChimeraX v1.8, DeepEMhancer version 2020.09.07 (https://github.com/rsanchezgarc/deepEMhancer); Model Building & Refinement: COOT v0.9.6, Phenix.refine v1.21.1, AlphaFold2; Sequence alignment: MUSCLE (as implemented in Snapgene v7.2); Biochemical Data Analysis: ImageJ 1.54, GraphPad Prism 10; Figure Generation: Adobe Illustrator 2024, ChemDraw 22.2.<br>All software used was either available commercially (CryoSPARC, Snapgene, GraphPad Prism, Adobe Illustrator, ChemDraw) or as open source (others). No custom code was generated or used for analyses in this study.

For manuscripts utilizing custom algorithms or software that are central to the research but not yet described in published literature, software must be made available to editors and reviewers. We strongly encourage code deposition in a community repository (e.g. GitHub). See the Nature Portfolio guidelines for submitting code & software for further information.

Data

Policy information about availability of data

All manuscripts must include a data availability statement. This statement should provide the following information, where applicable:

- Accession codes, unique identifiers, or web links for publicly available datasets
- A description of any restrictions on data availability
- For clinical datasets or third party data, please ensure that the statement adheres to our policy

The structural data will be available from EMDB and RCSB upon manuscript publication (TRIP12deltaN branched K29/K48-linked chain formation: EMD-51429, PDB 9GKM; TRIP12deltaN K29-linked di-ubiquitin formation: EMD-51430, PDB 9GKN). Cryo-EM map were deposited and are available via the following accession number: TRIP12 FL branched K29/K48-linked chain formation: EMD-51428.

Research involving human participants, their data, or biological material

Policy information about studies with human participants or human data. See also policy information about sex, gender (identity/presentation), and sexual orientation and race, ethnicity and racism.

Reporting on sex and gender	No research involving human participants has been performed.
Reporting on race, ethnicity, or other socially relevant groupings	No research involving human participants has been performed.
Population characteristics	No research involving human participants has been performed.
Recruitment	No research involving human participants has been performed.
Ethics oversight	No research involving human participants has been performed.

Note that full information on the approval of the study protocol must also be provided in the manuscript.

Field-specific reporting

Please select the one below that is the best fit for your research. If you are not sure, read the appropriate sections before making your selection.

☒ Life sciences ☐ Behavioural & social sciences ☐ Ecological, evolutionary & environmental sciences

For a reference copy of the document with all sections, see nature.com/documents/nr-reporting-summary-flat.pdf

Life sciences study design

All studies must disclose on these points even when the disclosure is negative.

Sample size	Sample size calculations were not performed. Selected sample sizes were designed to ensure clear and reliable interpretation of the results. Based on previous experience in terms of variability, at least two independent replicates were carried out for all functional assays, as is standard for such experiments for other studies in this field (e.g. refs 9 & 10).
Data exclusions	No data were excluded.
Replication	All biochemical experiments were performed (at least) in technical duplicates along with appropriate controls, with successful replication of results in all cases.
Randomization	No grouped samples.
Blinding	No grouped samples.

Reporting for specific materials, systems and methods

We require information from authors about some types of materials, experimental systems and methods used in many studies. Here, indicate whether each material, system or method listed is relevant to your study. If you are not sure if a list item applies to your research, read the appropriate section before selecting a response.

Materials & experimental systems

n/a	Involved in the study
☐	☒ Antibodies
☐	☒ Eukaryotic cell lines
☒	☐ Palaeontology and archaeology
☒	☐ Animals and other organisms
☒	☐ Clinical data
☒	☐ Dual use research of concern
☒	☐ Plants

Methods

n/a	Involved in the study
☒	☐ ChIP-seq
☒	☐ Flow cytometry
☒	☐ MRI-based neuroimaging

Antibodies

Antibodies used

Anti-Ubiquitin (P4D1) mouse mAb (HRP conjugate), Cell Signaling Technology, Cat. No. 14049S, Lot 3; 1:5000 dilution

Validation

https://www.cellsignal.com/products/antibody-conjugates/ubiquitin-p4d1-mouse-mab-hrp-conjugate/14049

Eukaryotic cell lines

Policy information about cell lines and Sex and Gender in Research

Cell line source(s)

HEK293S GnTI- (identifier: CRL-3022) were obtained from ATCC. High five cells (BTI-TN-5B1-4) were obtained from ThermoFisher Scientific (catalogue number:B85502). Gibco Sf9 cells were obtained from ThermoFisher Scientific (catalogue number:11496016).

Authentication

Cell lines were not authenticated.

Mycoplasma contamination

Cell lines were periodically tested for mycoplasm contamination with no contamination detected.

Commonly misidentified lines
(See ICLAC register)

No commonly misidentified cell lines were used in this study.

Plants

Seed stocks

Report on the source of all seed stocks or other plant material used. If applicable, state the seed stock centre and catalogue number. If plant specimens were collected from the field, describe the collection location, date and sampling procedures.

Novel plant genotypes

Describe the methods by which all novel plant genotypes were produced. This includes those generated by transgenic approaches, gene editing, chemical/radiation-based mutagenesis and hybridization. For transgenic lines, describe the transformation method, the number of independent lines analyzed and the generation upon which experiments were performed. For gene-edited lines, describe the editor used, the endogenous sequence targeted for editing, the targeting guide RNA sequence (if applicable) and how the editor was applied.

Authentication

Describe any authentication procedures for each seed stock used or novel genotype generated. Describe any experiments used to assess the effect of a mutation and, where applicable, how potential secondary effects (e.g. second site T-DNA insertions, mosiacism, off-target gene editing) were examined.
