# Supplementary figures and images for: TRIP12 structures reveal HECT E3 formation of K29 linkages and branched ubiquitin chains
Source: Nat Struct Mol Biol. 2025 May 26;32(9):1766–75. doi: 10.1038/s41594-025-01561-1 (PMC12440805; doi:10.1038/s41594-025-01561-1)

Fig. 1a

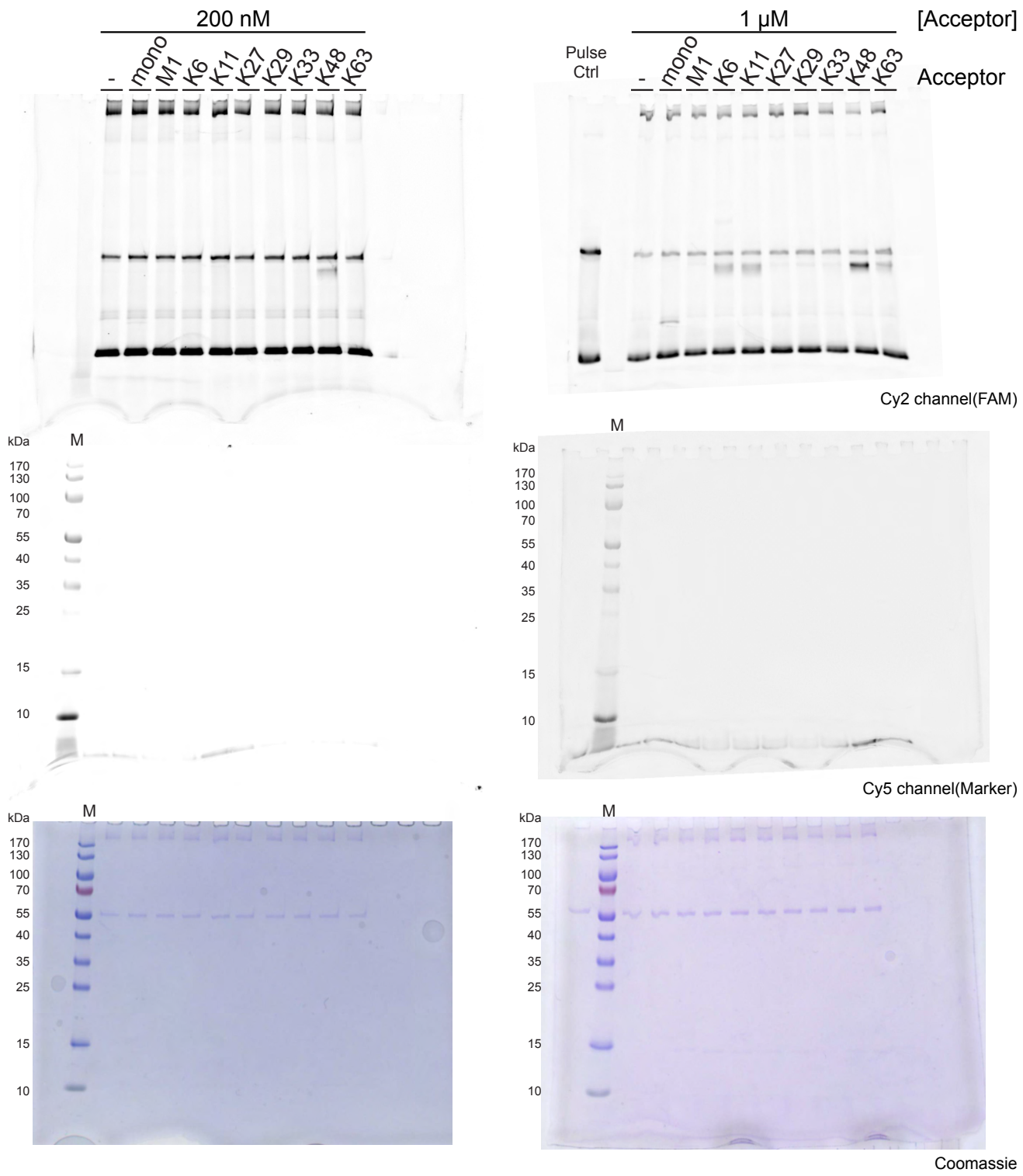

Fig. 1b

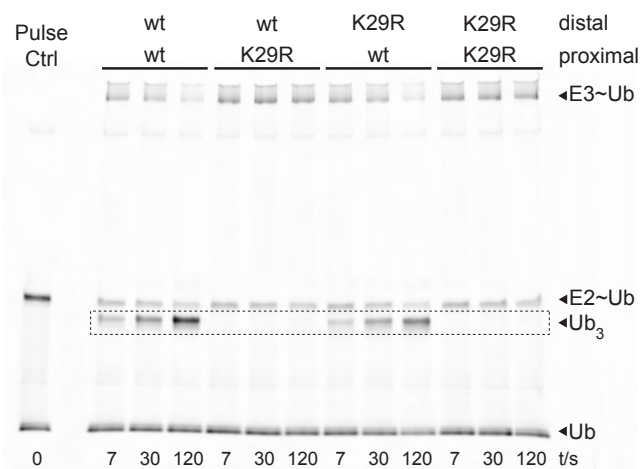

Cy2 channel(FAM)

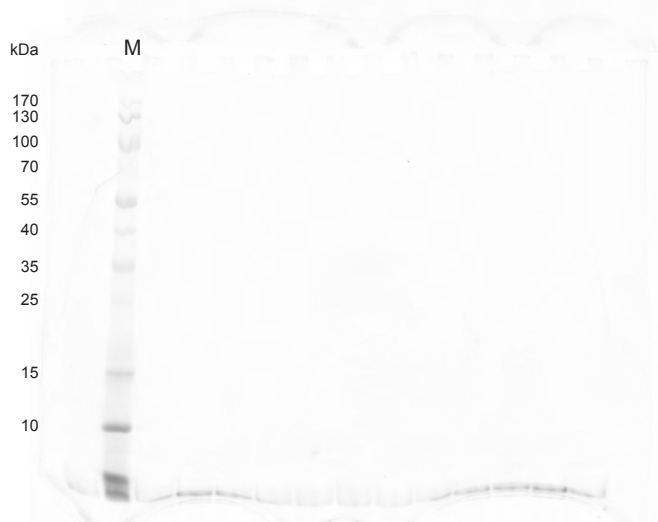

Cy5 channel(Marker)

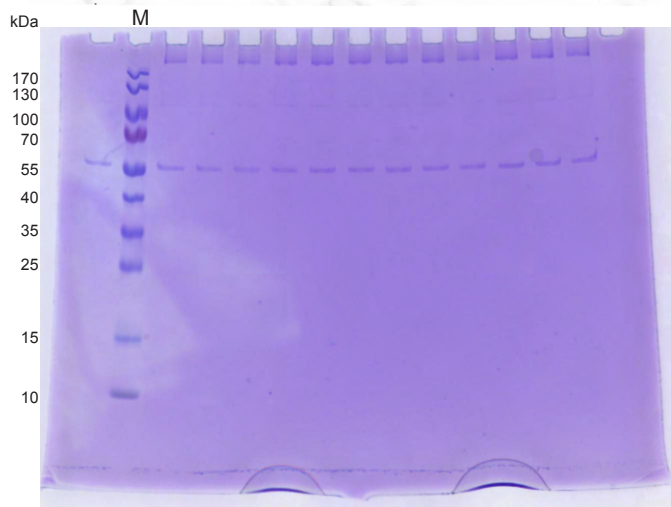

Coomassie

Fig. 1c - representative gel

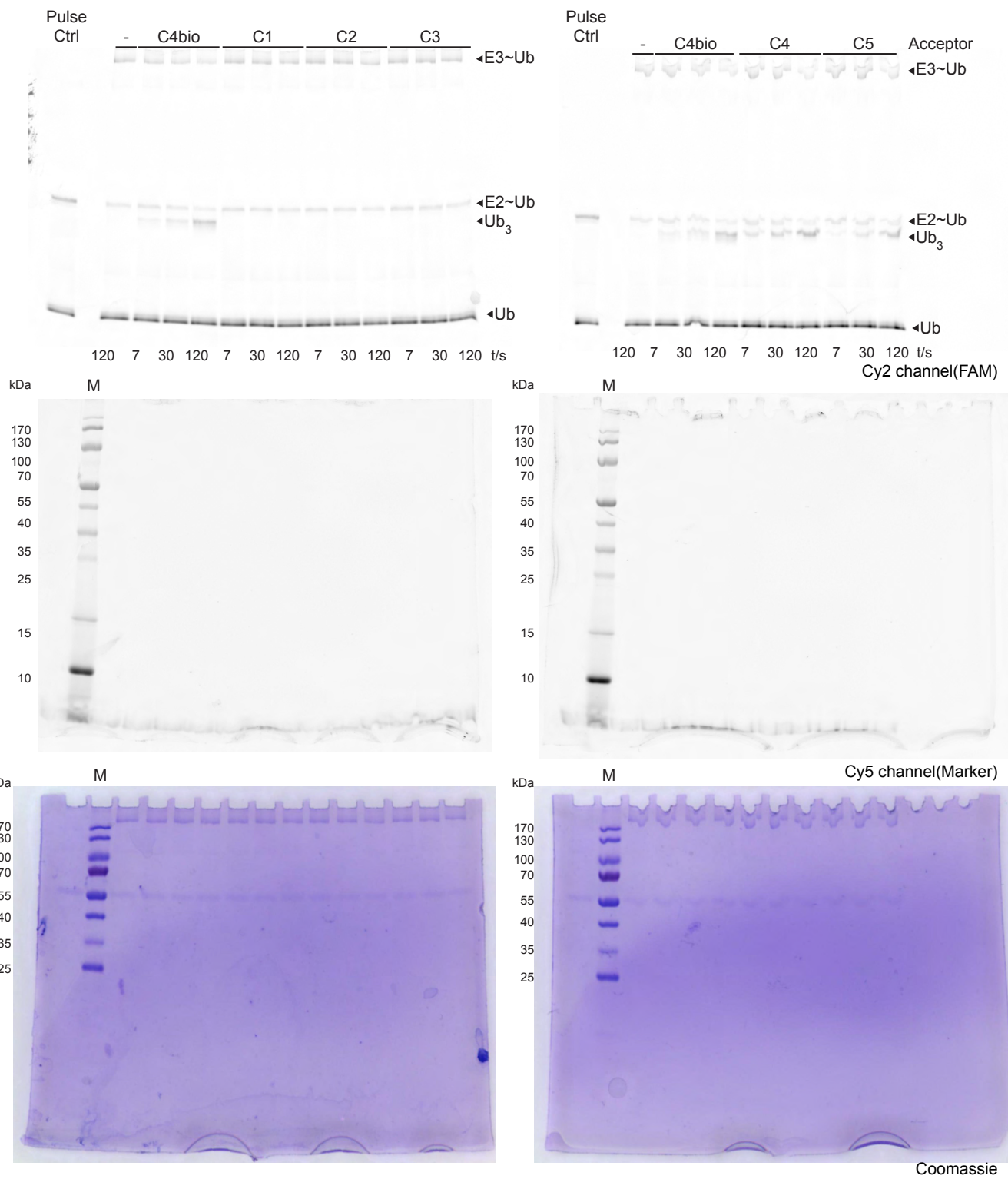

Supplement: Supplementary file 7 — Unprocessed gel scans and Coomassie-stained gels. [file 41594_2025_1561_MOESM7_ESM.pdf]

Fig. 3a

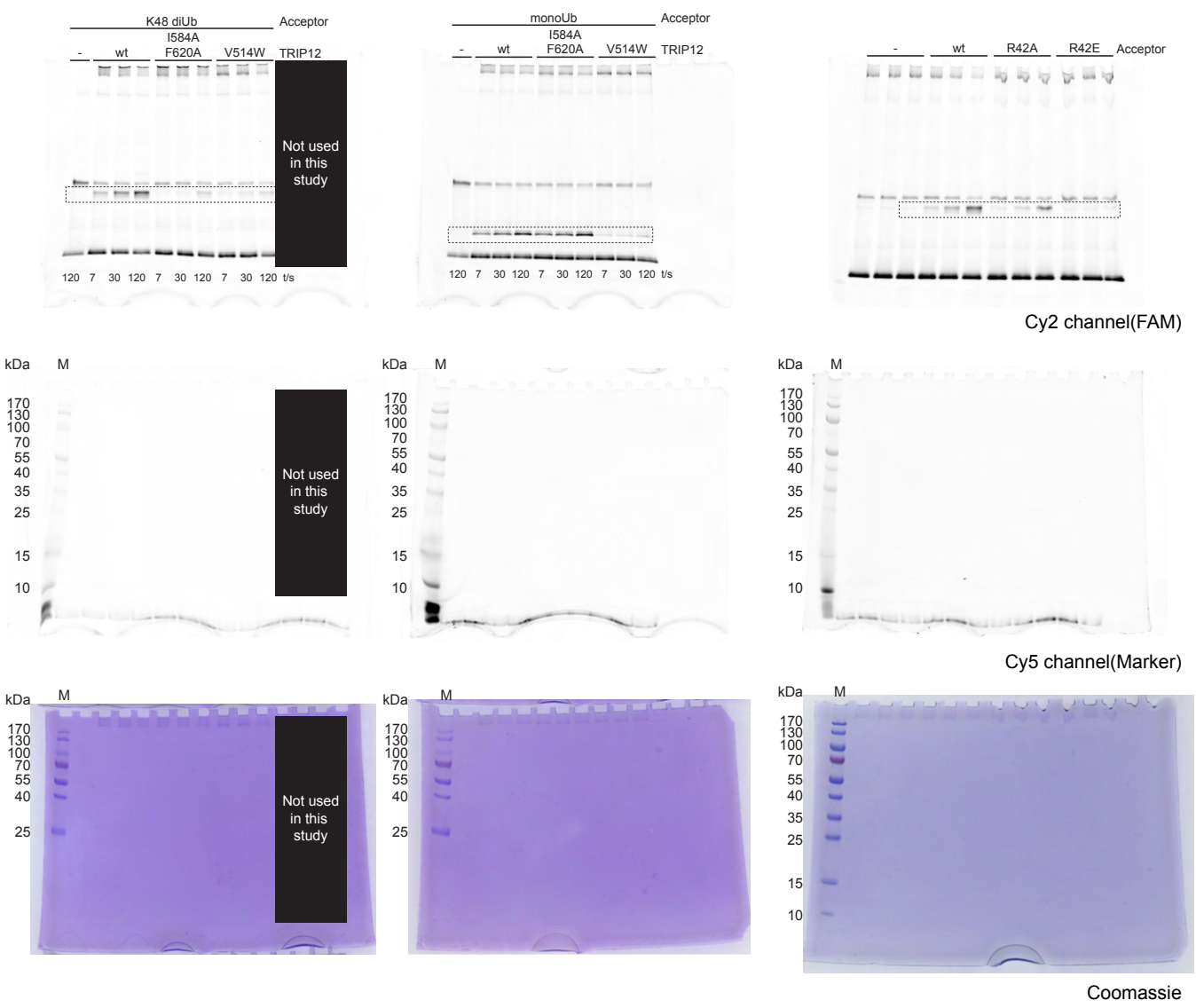

Supplement: Supplementary file 8 — Unprocessed gel scans and Coomassie-stained gels. [file 41594_2025_1561_MOESM8_ESM.pdf]

Fig. 5d, left panel; full gels

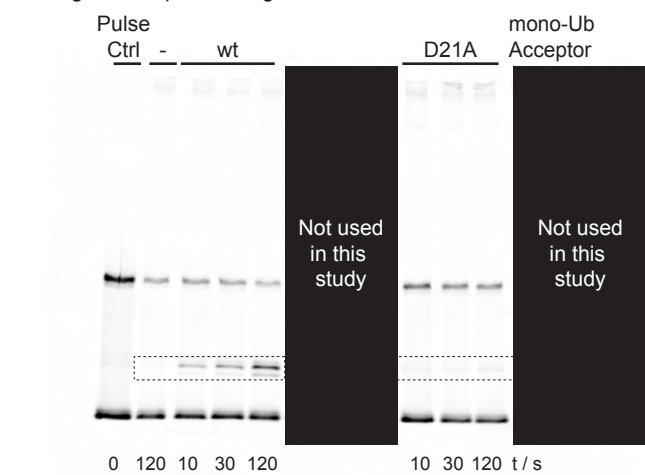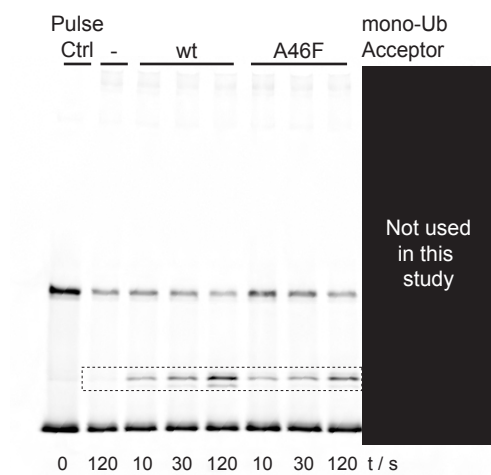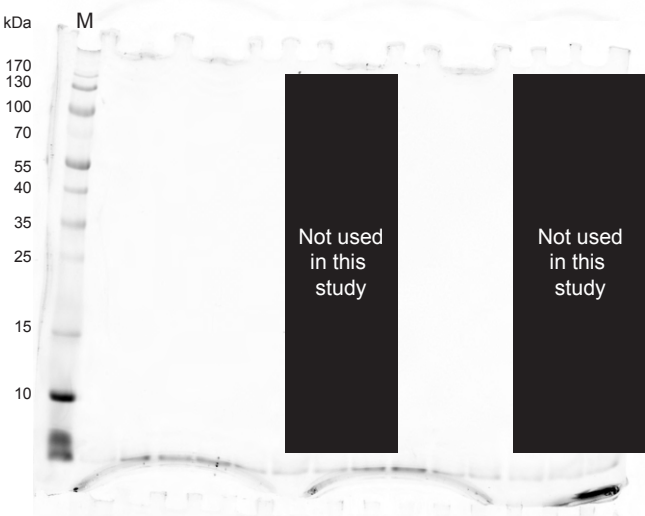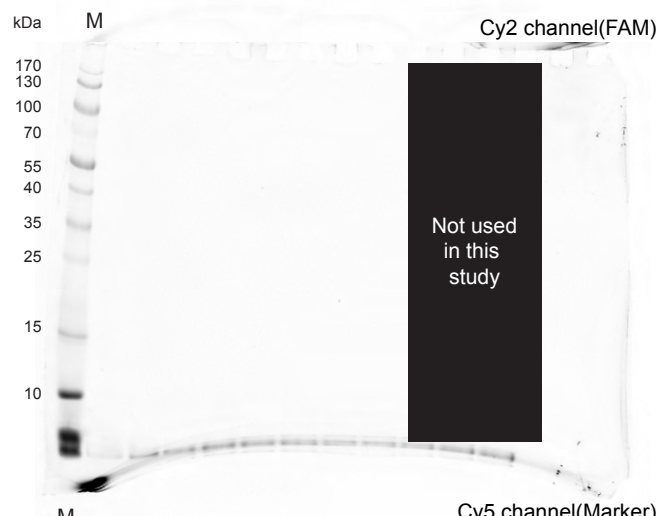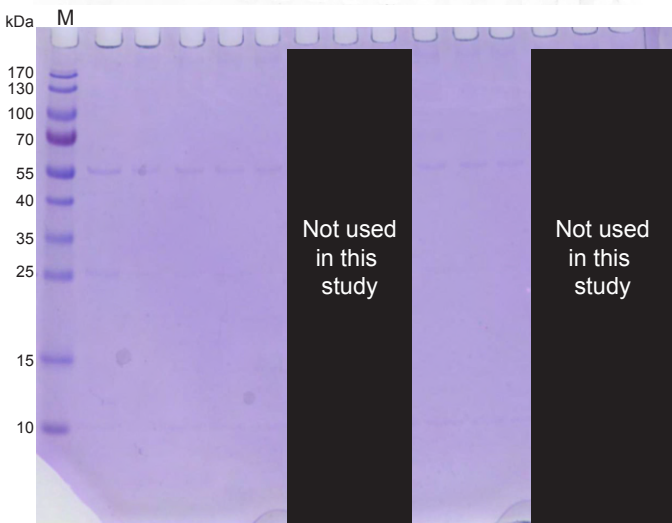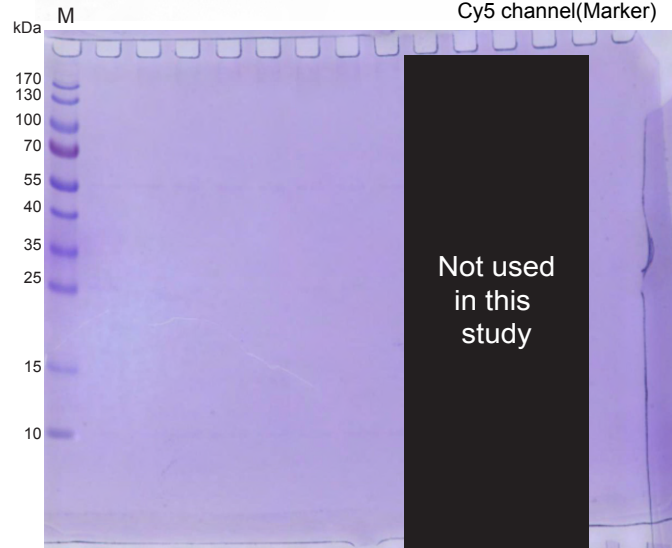

Fig. 5d, right panel; full gels

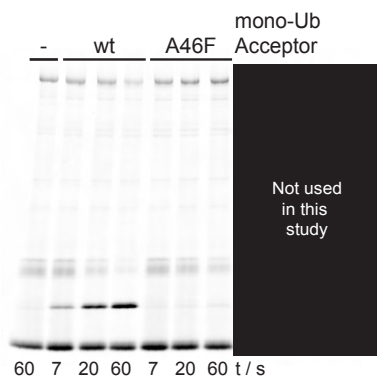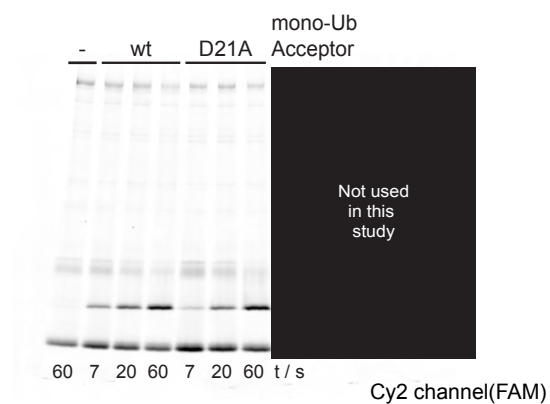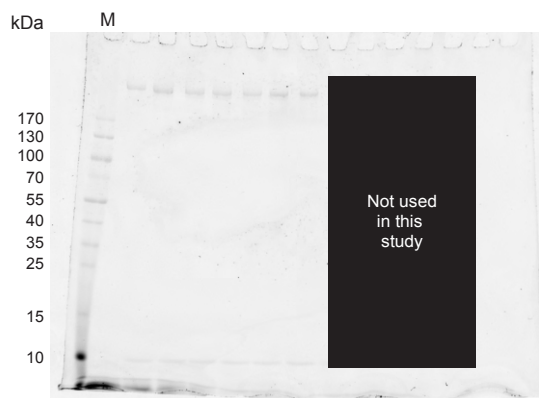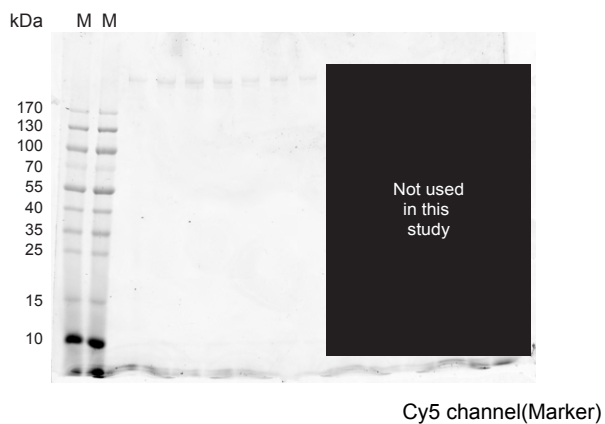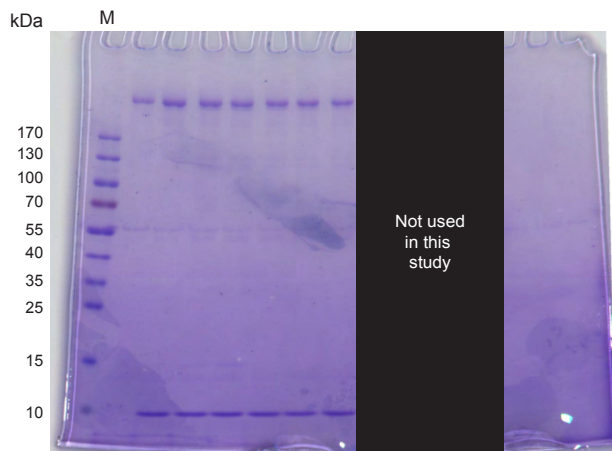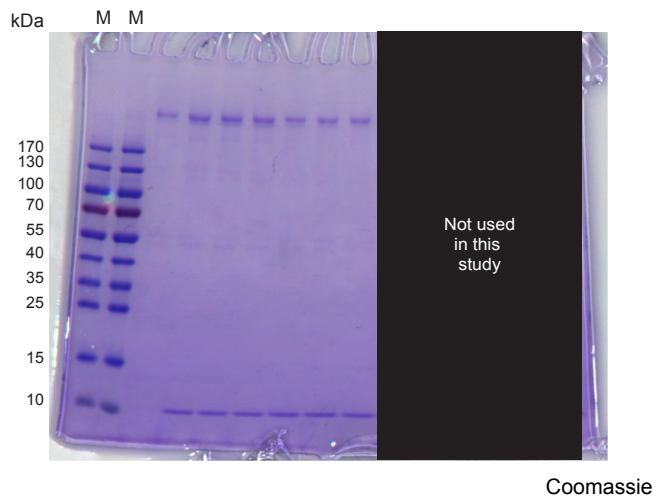

Supplement: Supplementary file 10 — Unprocessed gel scans and Coomassie-stained gels. [file 41594_2025_1561_MOESM10_ESM.pdf]

Extended Data Fig. 1a

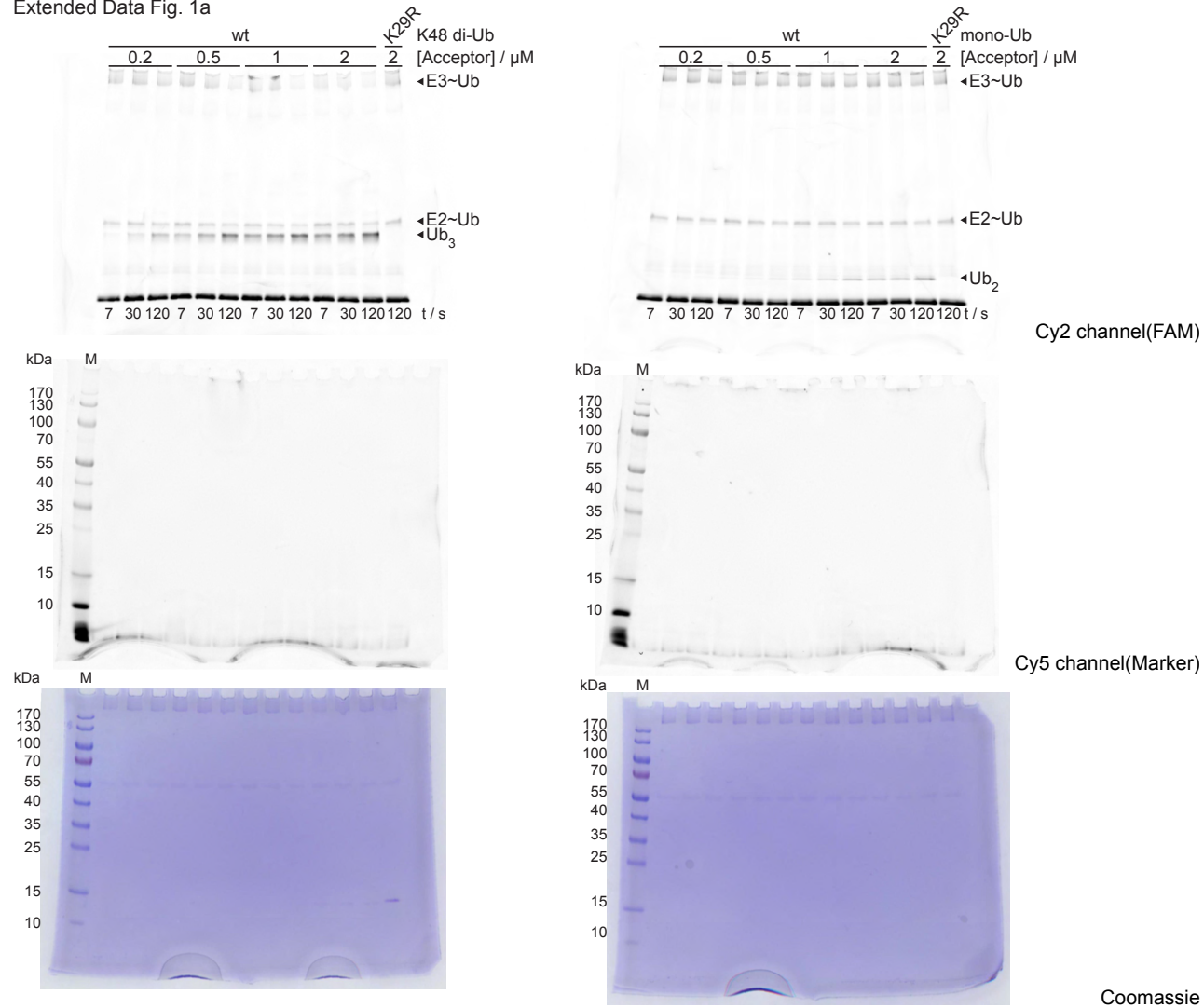

Extended Data Fig. 1b

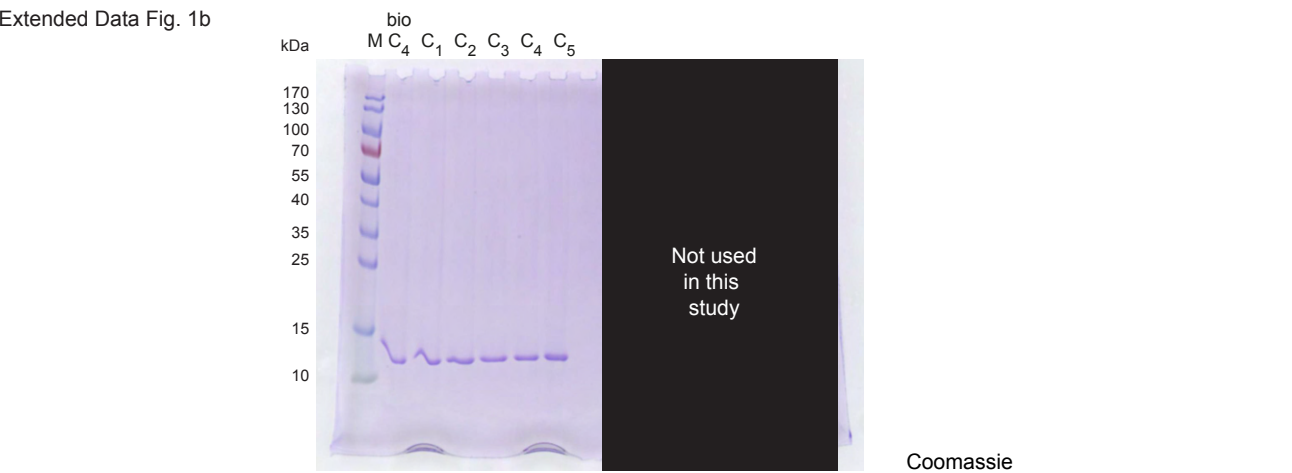

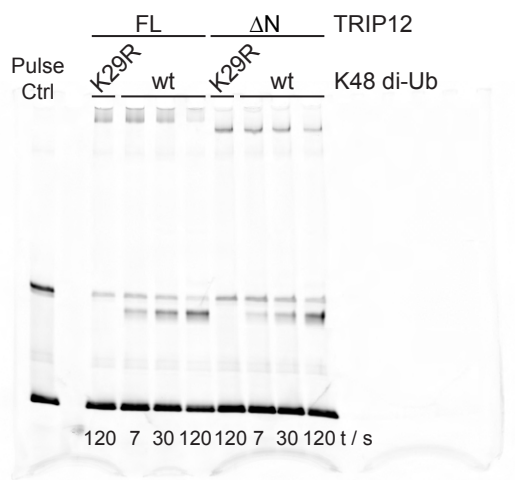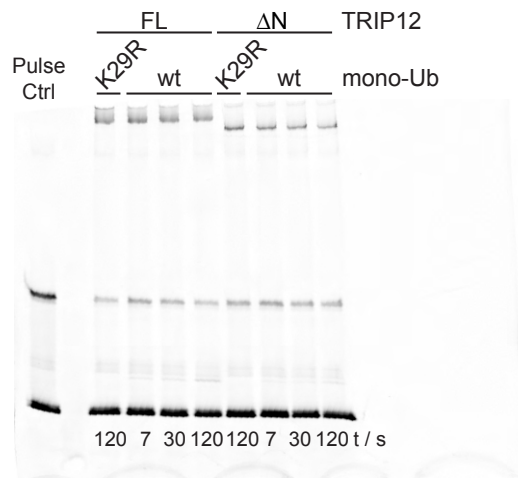

Cy2 channel(FAM)

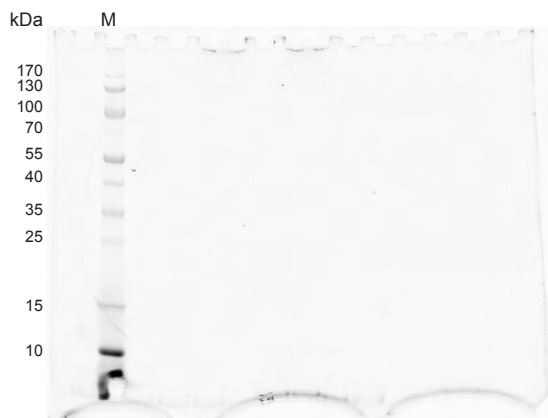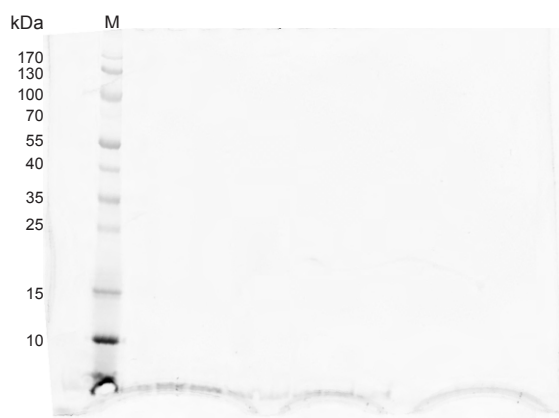

Cy5 channel(Marker)

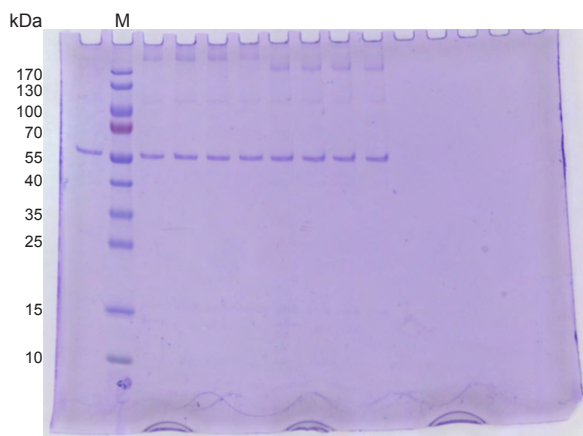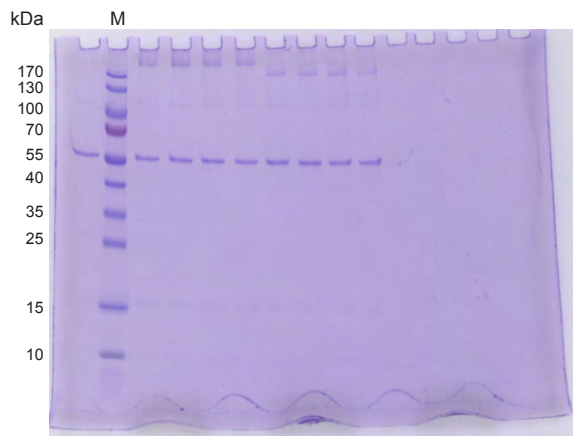

Coomassie

Supplement: Supplementary file 11 — Unprocessed gel scans and Coomassie-stained gels. [file 41594_2025_1561_MOESM11_ESM.pdf]

Extended Data Fig. 9a

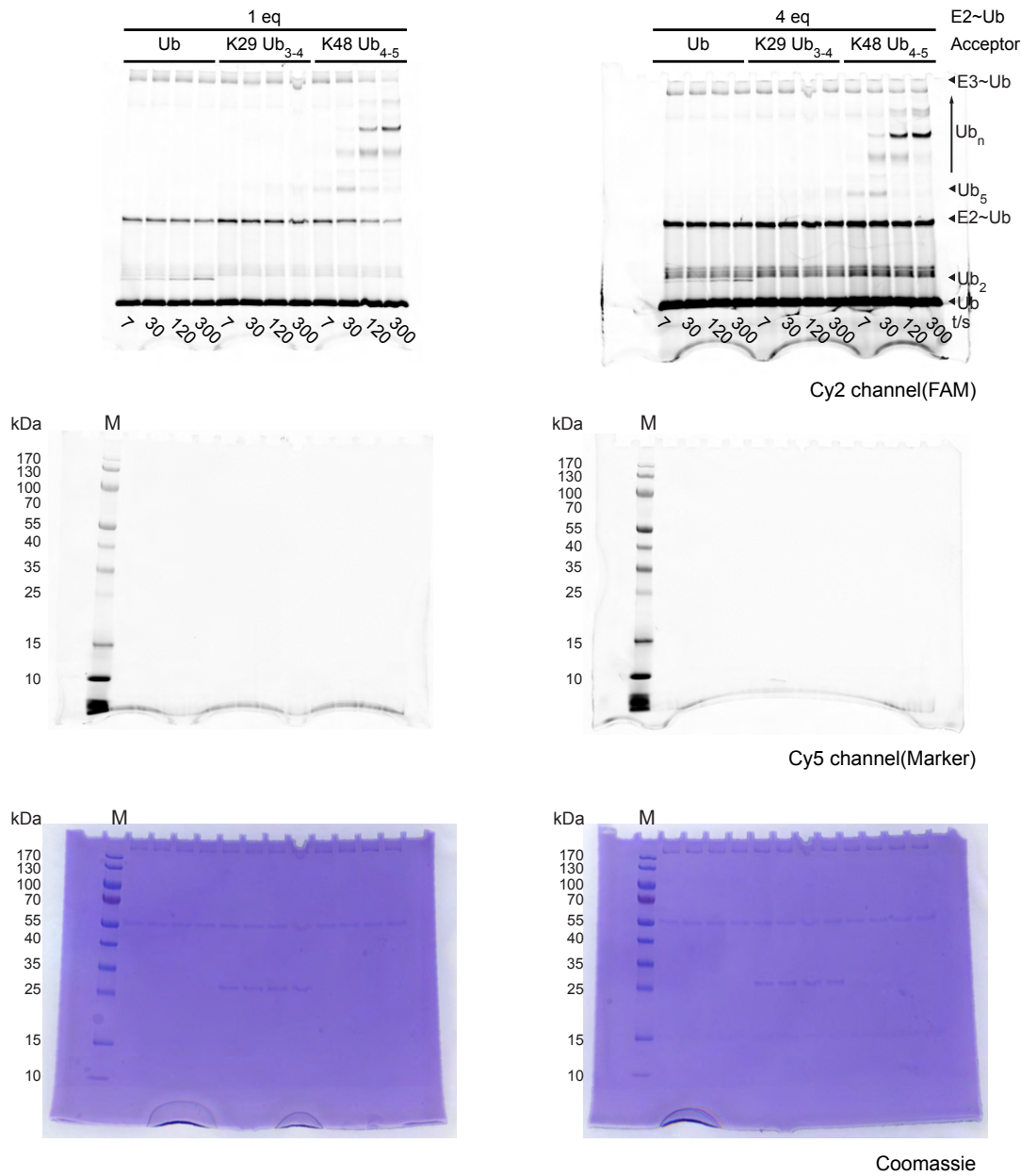

Extended Data Fig. 9b

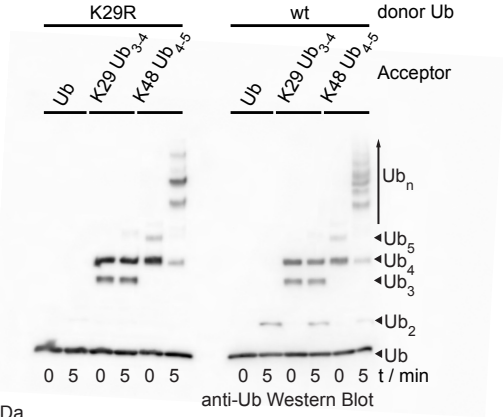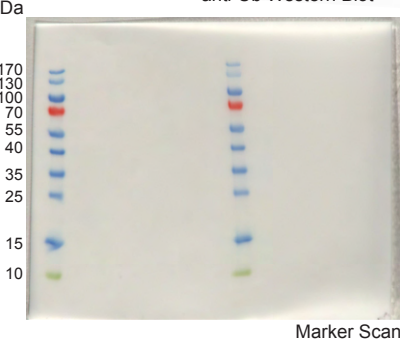

Extended Data Fig. 9c

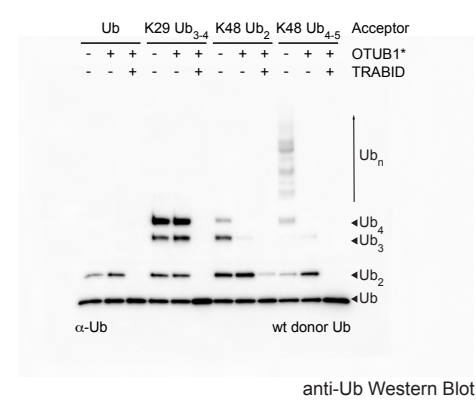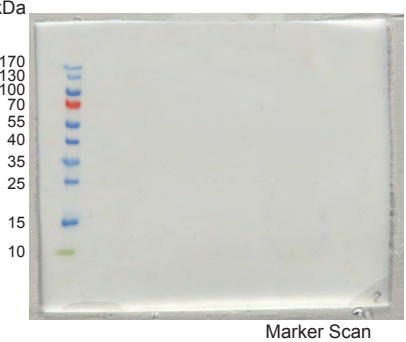

Supplement: Supplementary file 13 — Unprocessed gel scans, Coomassie-stained gels and unprocessed western blots. [file 41594_2025_1561_MOESM13_ESM.pdf]
