# Supplementary material for: TRIP12 structures reveal HECT E3 formation of K29 linkages and branched ubiquitin chains
Source: Nat Struct Mol Biol. 2025 May 26;32(9):1766–75. doi: 10.1038/s41594-025-01561-1 (PMC12440805; doi:10.1038/s41594-025-01561-1)

Fig. 4a, top right panel

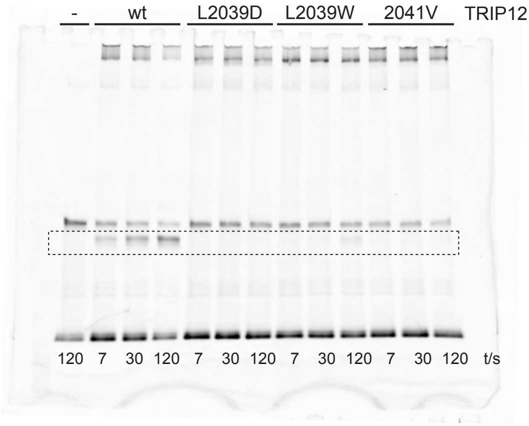

Fig. 4a,c; right bracket used in a (top center panel), center brackets in c

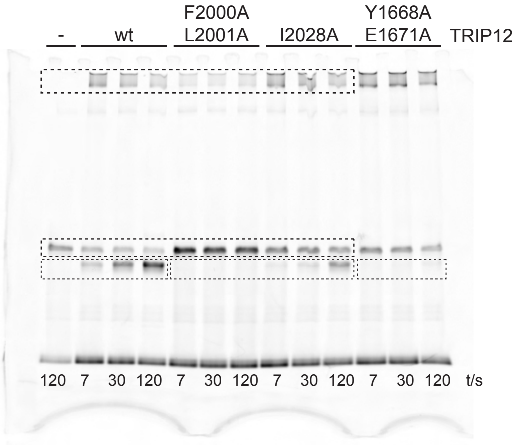

Cy2 channel(FAM)

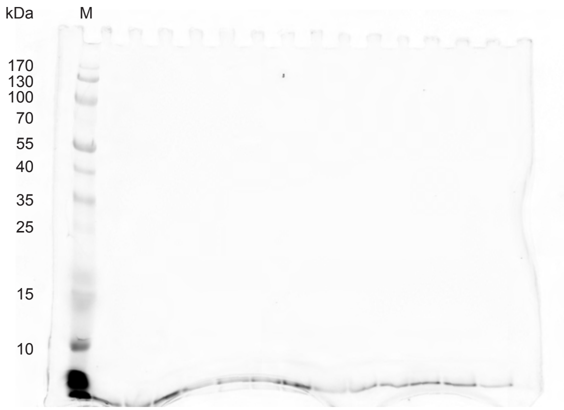

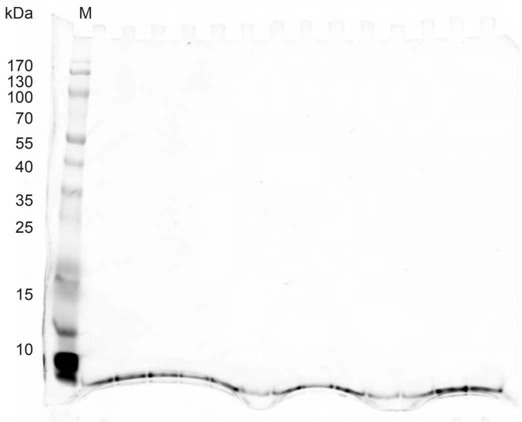

Cy5 channel(Marker)

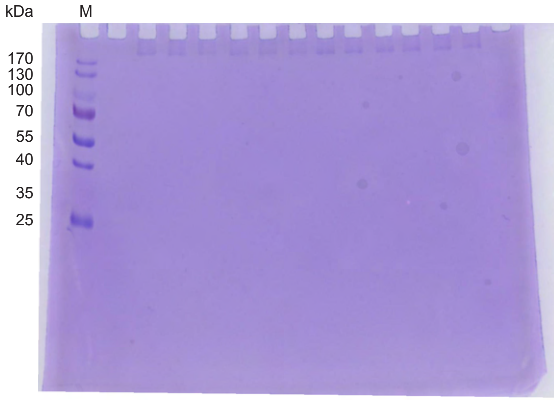

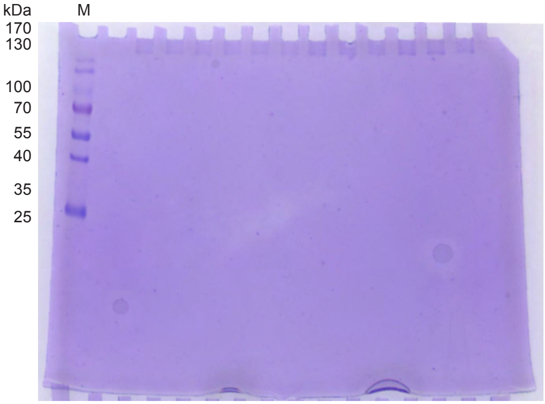

Coomassie

Fig. 4a, left center panel

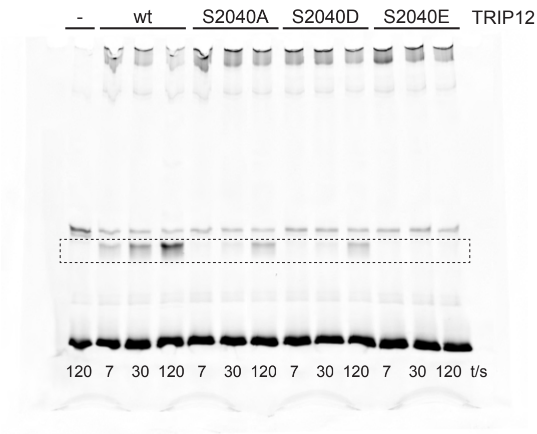

Fig. 4a, right center panel

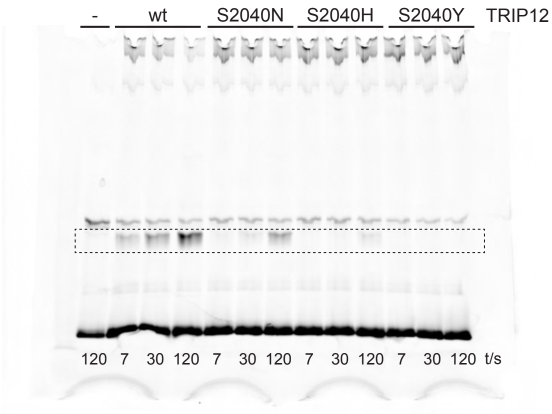

Cy2 channel(FAM)

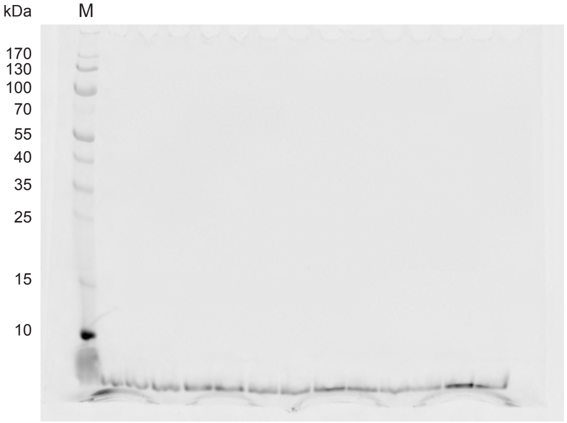

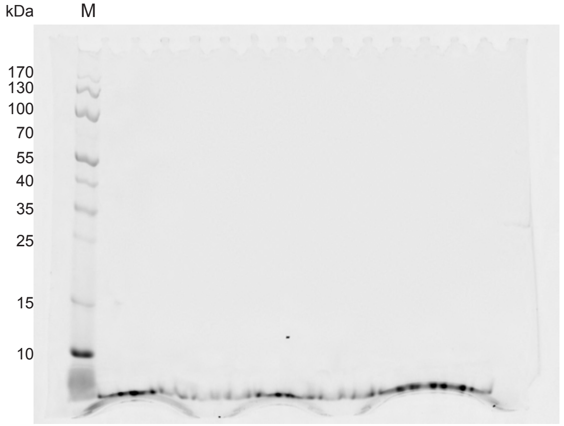

Cy5 channel(Marker)

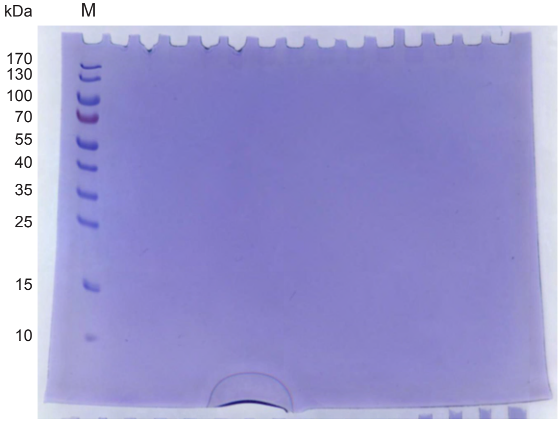

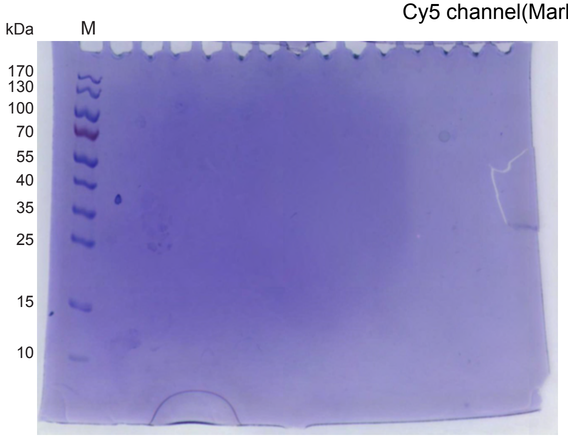

Coomassie

Fig. 4a, bottom panel

Fig. 4b, upper panel

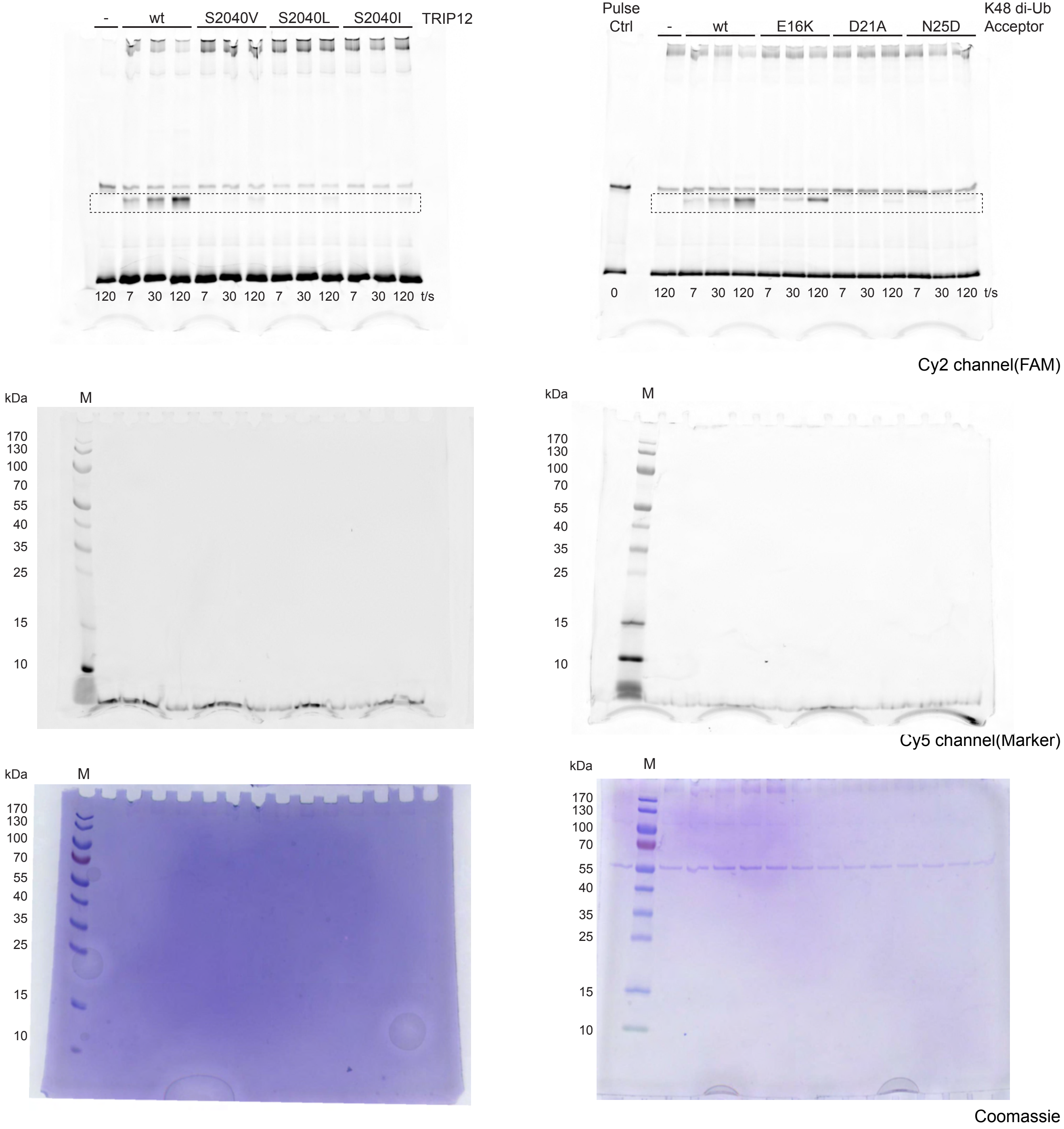

Fig. 4b, center and lower panel

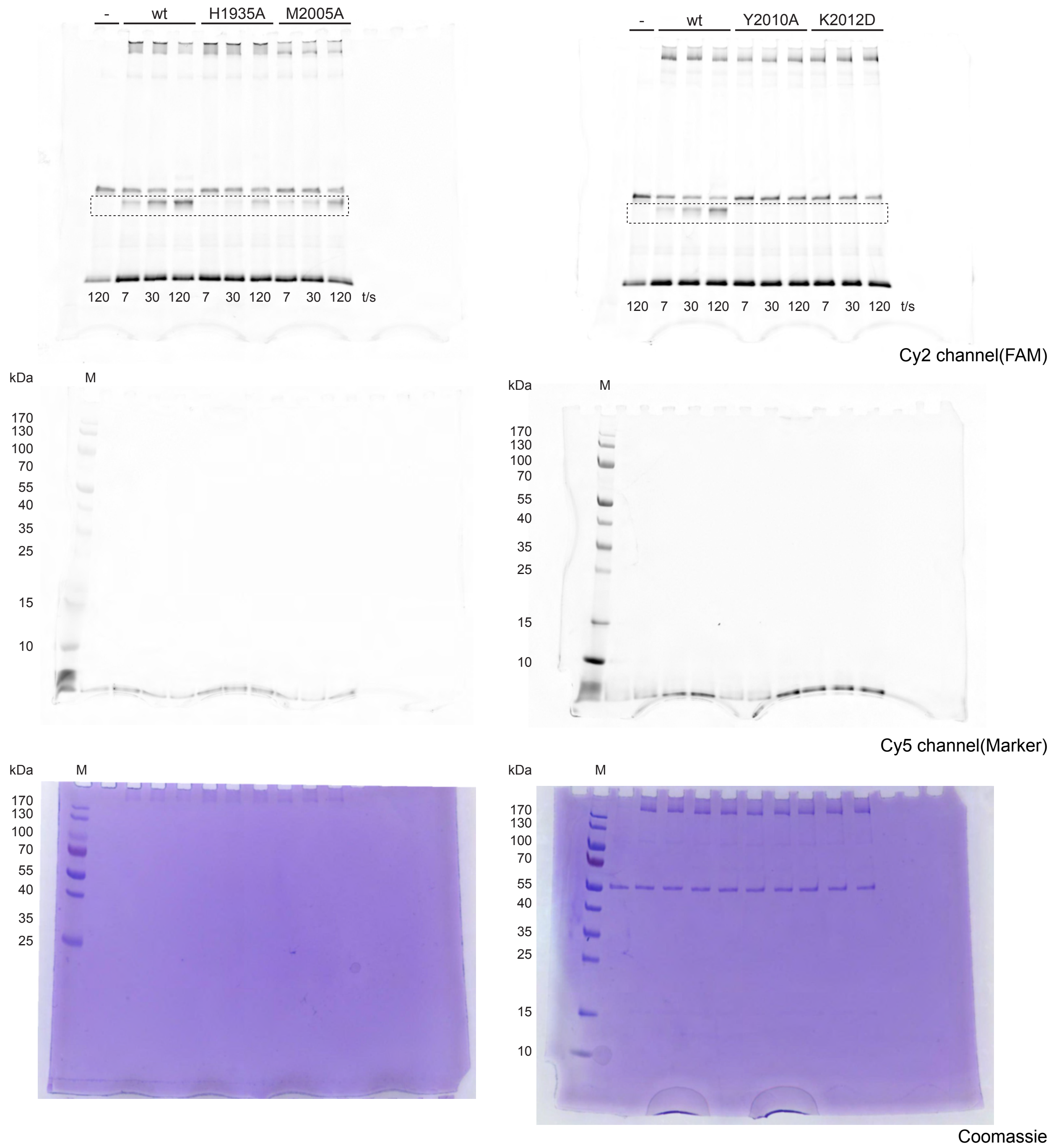

Supplement: Supplementary file 9 — Unprocessed gel scans and Coomassie-stained gels. [file 41594_2025_1561_MOESM9_ESM.pdf]
