# Supplementary material for: TRIP12 structures reveal HECT E3 formation of K29 linkages and branched ubiquitin chains
Source: Nat Struct Mol Biol. 2025 May 26;32(9):1766–75. doi: 10.1038/s41594-025-01561-1 (PMC12440805; doi:10.1038/s41594-025-01561-1)

Extended Data Fig. 8a

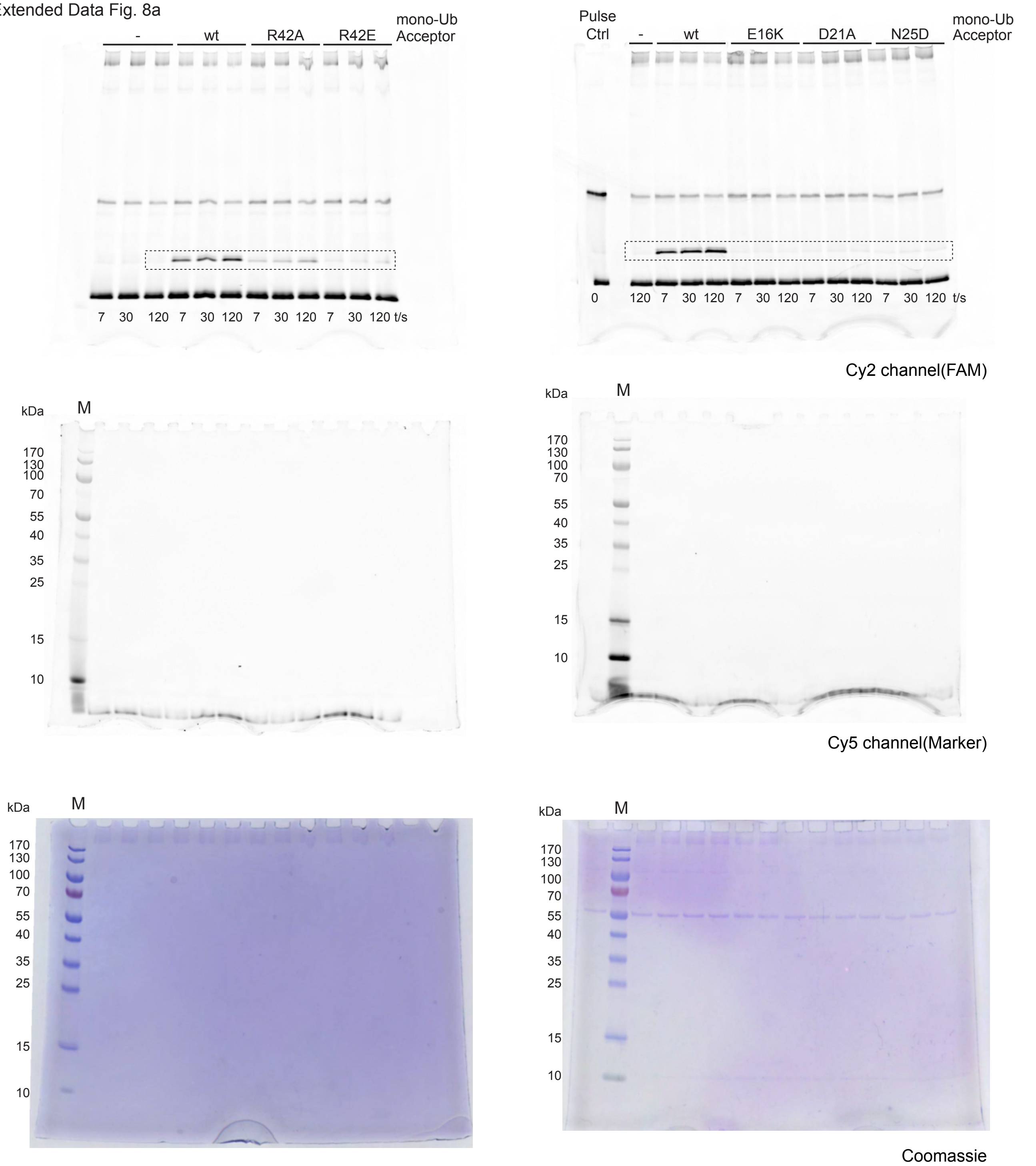

Extended Data Fig. 8a, continued

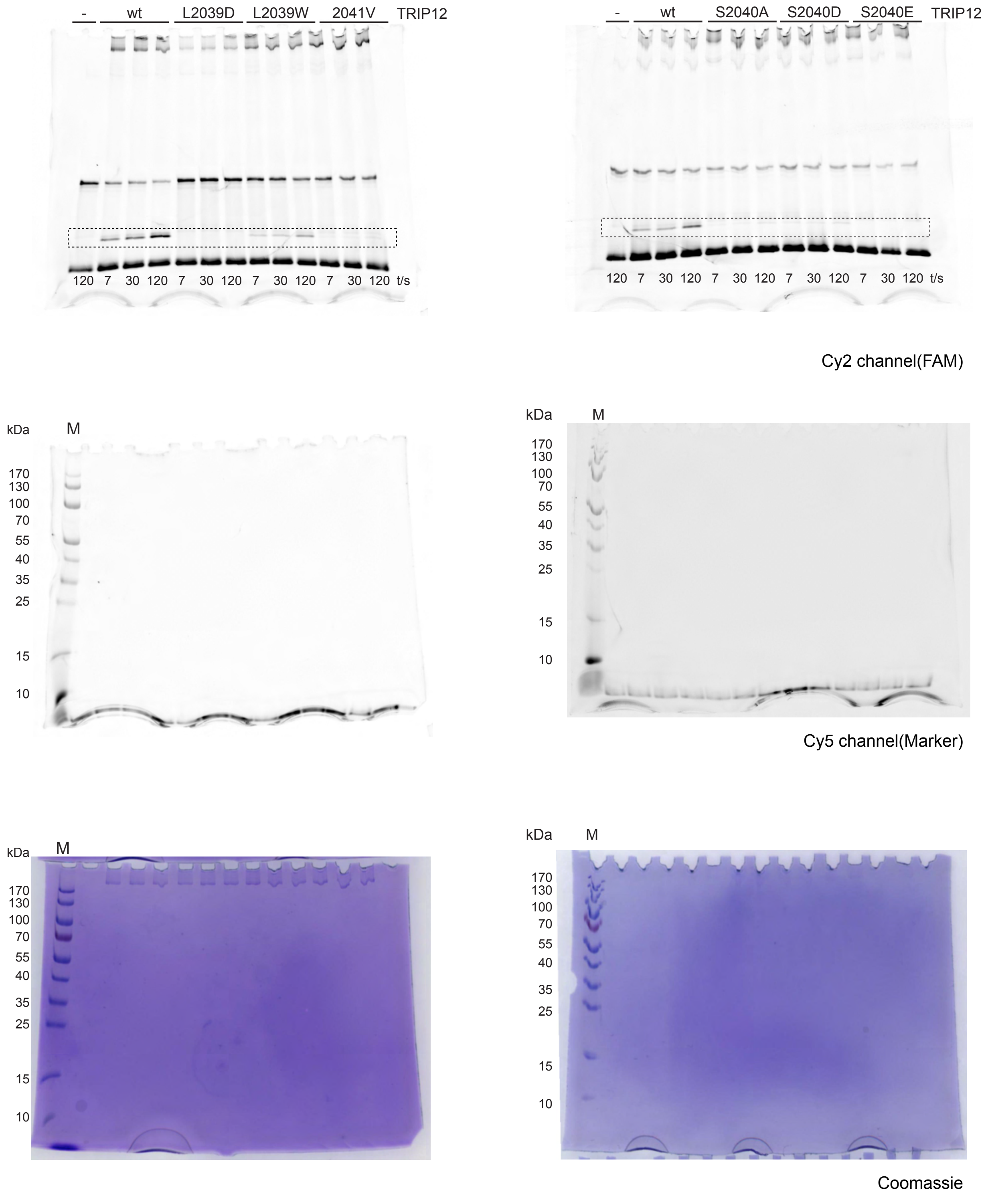

Extended Data Fig. 8a, continued

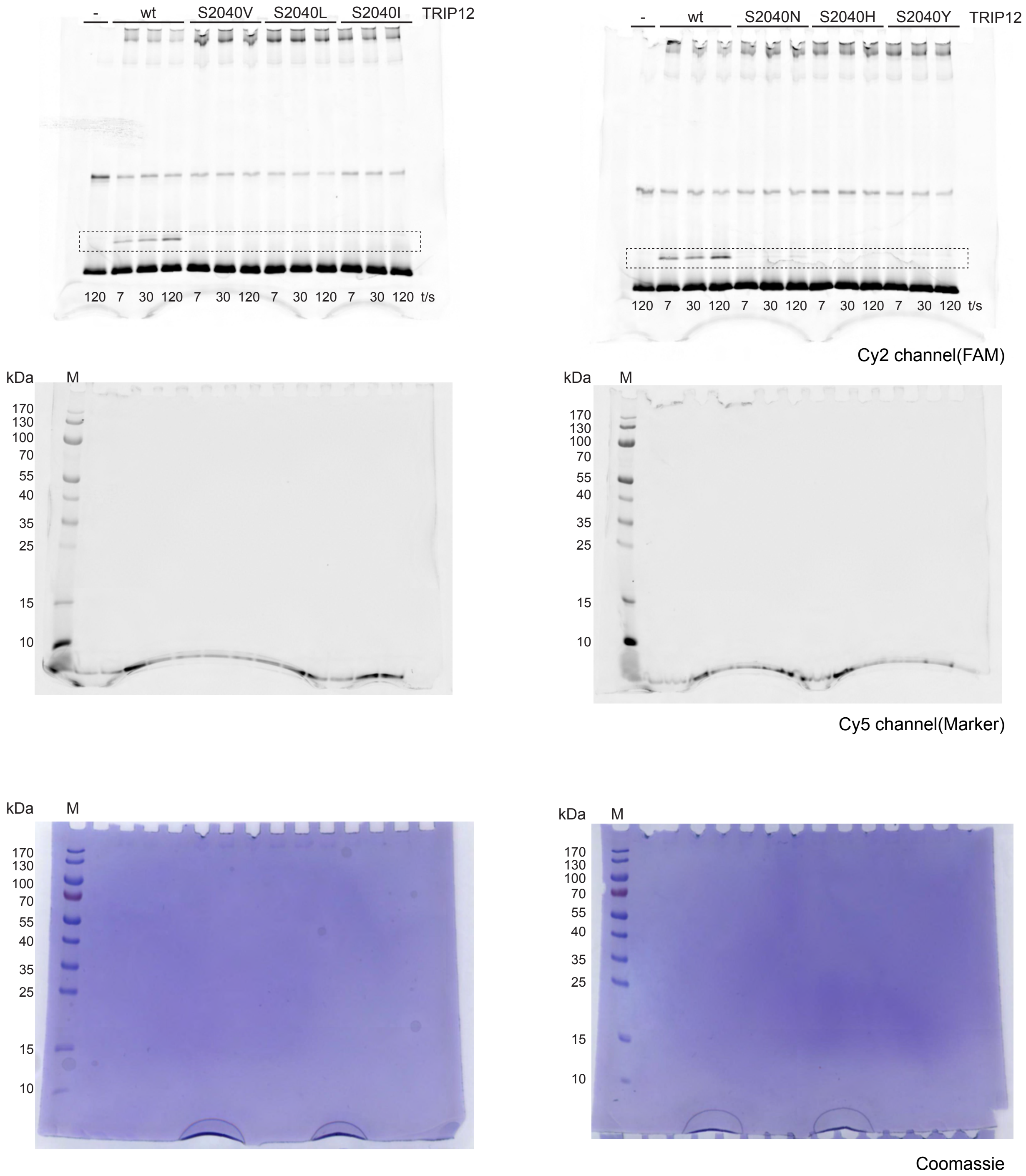

Extended Data Fig. 8a, continued

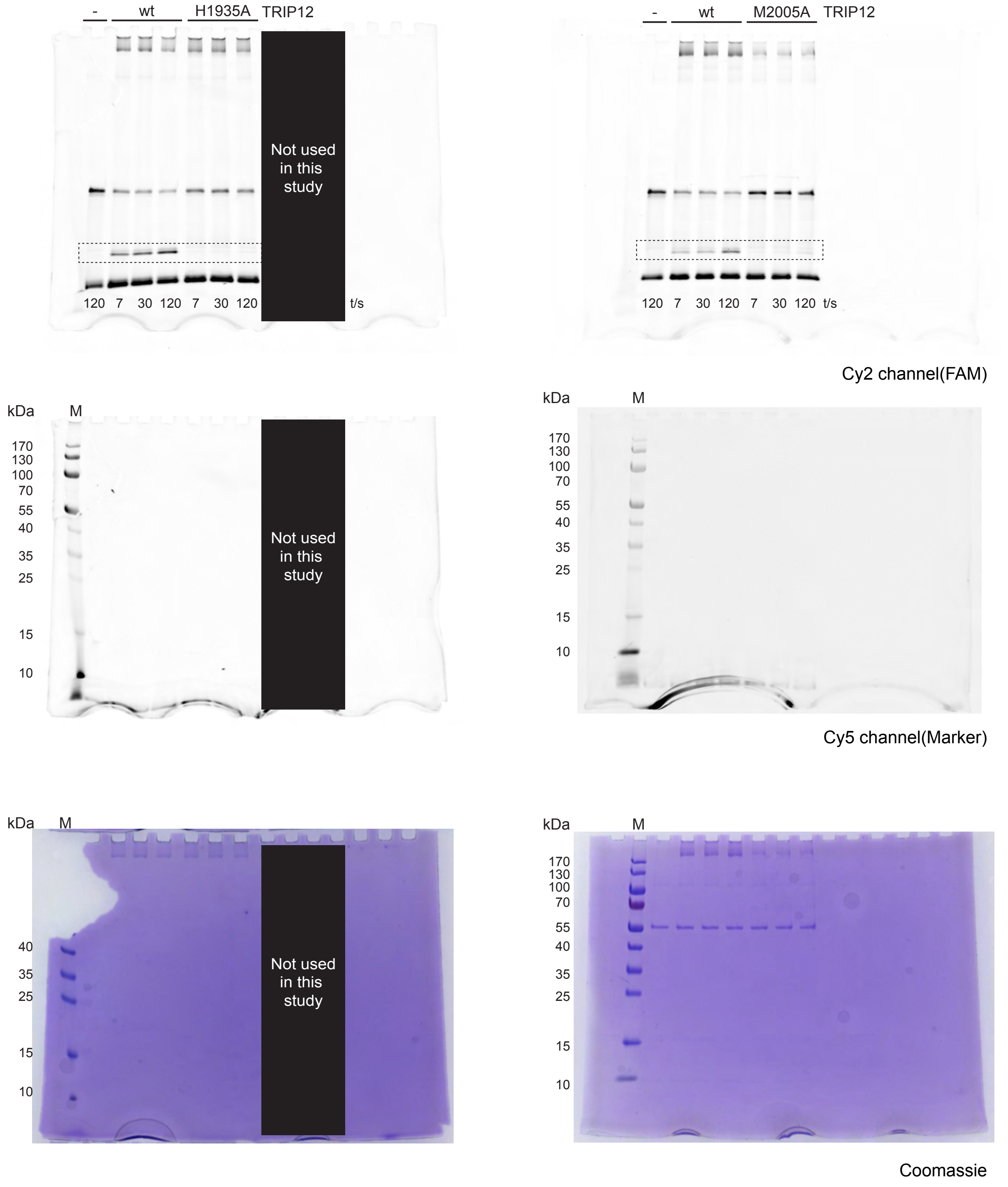

Extended Data Fig. 8a, continued

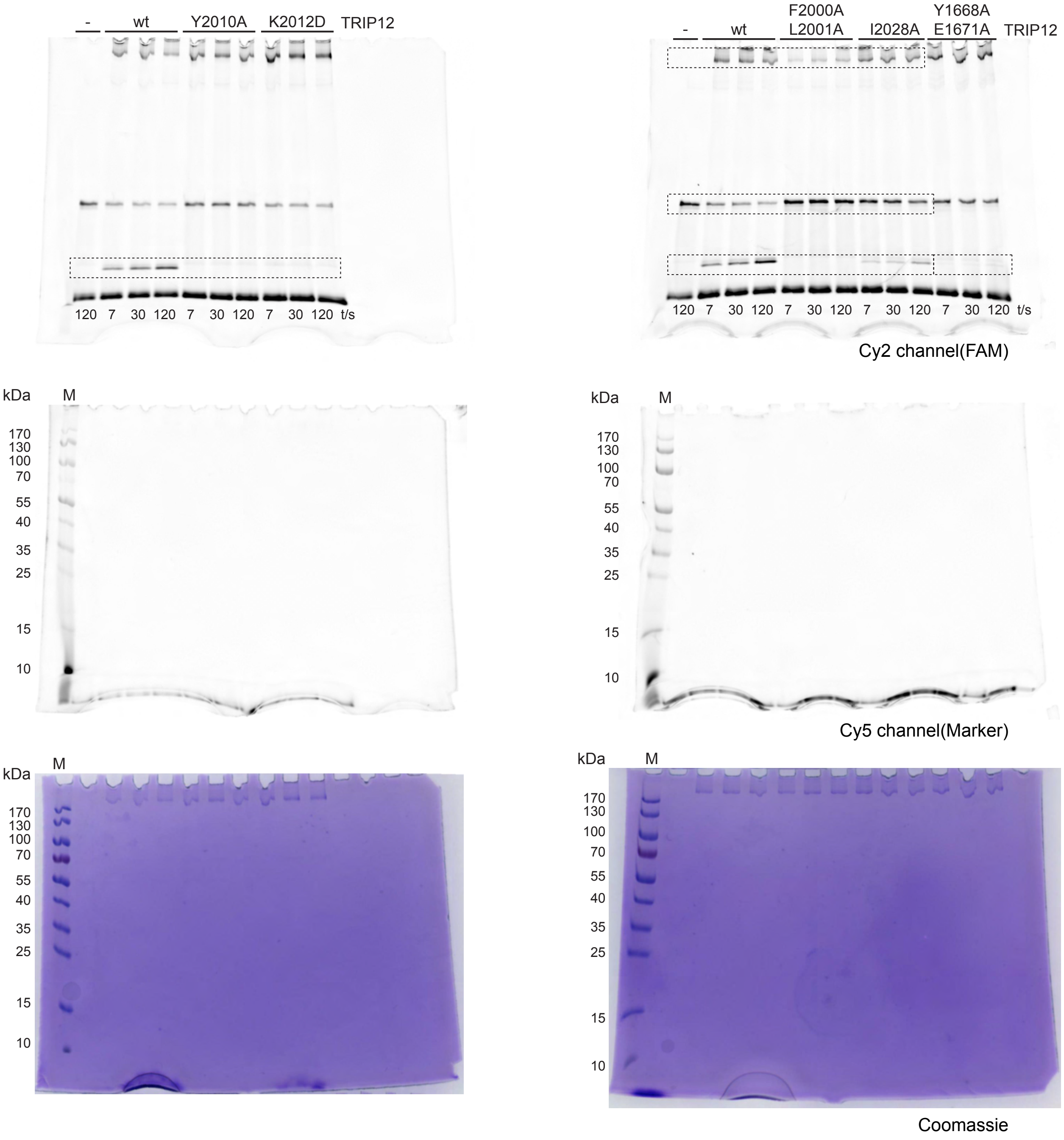

Supplement: Supplementary file 12 — Unprocessed gel scans and Coomassie-stained gels. [file 41594_2025_1561_MOESM12_ESM.pdf]
